# High-Throughput Silica Nanoparticle Detection for Quality Control of Complex Early Life Nutrition Food Matrices

**DOI:** 10.1021/acsomega.3c09459

**Published:** 2024-04-09

**Authors:** Viviana Maffeis, Andrea Otter, André Düsterloh, Lucy Kind, Cornelia Palivan, Sina S. Saxer

**Affiliations:** † University of Basel, Department of Chemistry, Mattenstrasse 22, 4002 Basel BS, Switzerland; ‡ NCCR-Molecular Systems Engineering, 4002 Basel, Switzerland; § DSM-Firmenich AG, Wurmisweg 576, 4313 Kaiseraugst AG, Switzerland; ∥ FHNW School of Life Sciences, Institute of Chemistry and Bioanalytics, Hofackerstrasse 30, 4132 Muttenz BL, Switzerland

## Abstract

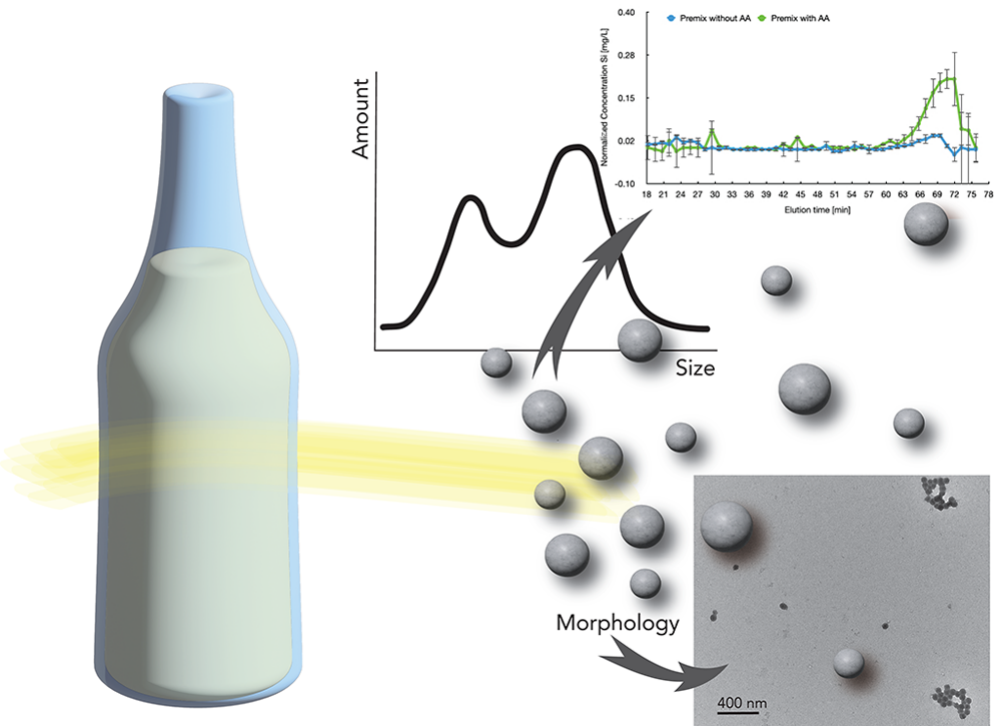

The addition of nanomaterials to improve product properties has become a matter of course for many commodities: e.g., detergents, cosmetics, and food products. While this practice improves product characteristics, the increasing exposure and potential impact of nanomaterials (<100 nm) raise concerns regarding both the human body and the environment. Special attention should be taken for vulnerable individuals such as those who are ill, elder, or newborns. But detecting and quantifying nanoparticles in complex food matrices like early life nutrition (ELN) poses a significant challenge due to the presence of additional particles, emulsion-droplets, or micelles. There is a pressing demand for standardized protocols for nanoparticle quantification and the specification of “nanoparticle-free” formulations. To address this, silica nanoparticles (SiNPs), commonly used as anticaking agents (AA) in processed food, were employed as a model system to establish characterization methods with different levels of accuracy and sensitivity versus speed, sample handling, and automatization. Different acid treatments were applied for sample digestion, followed by size exclusion chromatography. Morphology, size, and number of NPs were measured by transmission electron microscopy, and the amount of Si was determined by microwave plasma atomic emission spectrometry. This successfully enabled distinguishing SiNP content in ELN food formulations with 2–4% AA from AA-free formulations and sorting SiNPs with diameters of 20, 50, and 80 nm. Moreover, the study revealed the significant influence of the ELN matrix on sample preparation, separation, and characterization steps, necessitating method adaptations compared to the reference (SiNP in water). In the future, we expect these methods to be implemented in standard quality control of formulation processes, which demand high-throughput analysis and automated evaluation.

## Introduction

The use of nanoparticles has a long history, but it was only with the advancement of high-resolution microscopes that their characterization became possible, paving the way for the development of engineered nanoparticles designed for defined purposes. From 2007 on the use of nanomaterials in consumer goods, e.g., cosmetics, additives, clothing, detergents, electronics, automotives etc., increased tremendously.^1^ The most common used NPs are minerals, e.g., TiO_2_, SiO_2_, etc., and metals, e.g., Ag and Au, but also polymers (nanoplastics). The topic of nanomaterials (e.g., nanoplastics and nanoparticles) has just surfaced and demands new characterization methods. However, the regulatory framework has been slow to catch up, frequently focusing on the material itself and neglecting the changes in properties that arise from their nanoscale nature.^2^

Growing concerns about diseases potentially triggered by nanoparticles (NPs) have brought previously considered harmless nanomaterials, such as synthetic amorphous silica food additives (SAS or E551), back in focus of the European Food Safety Authority (EFSA).^3^ While the effect of such NPs is again under investigation, food engineering companies already reacted and abstained from NPs in food additives.^4^ For example, until 2009, the regulation of silicates and silicon dioxides in food additives was based on a 1974 WHO/FAO report indicating an acceptable daily intake as “not limited”. It was considered that silicates do not accumulate toxically, are excreted through urine, and thus appear to be biologically inert.^5^ In 2009 a fixed limit of 1500 mg of SiO_2_/day was established and confirmed by the EFSA as having no safety concerns.^6^ However, none of these reports considered the dimensions of silicates. Thus, nanoparticles, defined as particles with external dimensions of 1–100 nm (ERA nanomaterials in food/feed chain),^7^ were considered to react similarly to natural silicates. In 2018, the EFSA reevaluated the topic and concluded that EU specifications were insufficient to adequately characterize SiO_2_ as a food additive.^8^ The latest environmental risk assessment of the application of nanoscience and nanotechnology in the food and feed chain, presented in 2020, addresses the uptake and fate of behavior of engineered nanomaterials.^6^ The latest guidance of nanoscience and nanotechnology in the food and feed chain (2020) categorizes materials as nanomaterials if they contain more than 10% of particles measuring below 500 nm.^9^ In addition, a threshold for NP size of 250 nm was defined, which relates to the material’s potential uptake by the gastrointestinal tract and determines whether it qualifies as a nanoengineered nanomaterial or not. For an adequate count of NPs, a proper sample dispersion and electron microscopy are recommended. However, only a few standard operating procedures (SOPs) are available as yet, e.g. steel, kaolin, TiO_2_, etc.^10^

**Figure 1:**
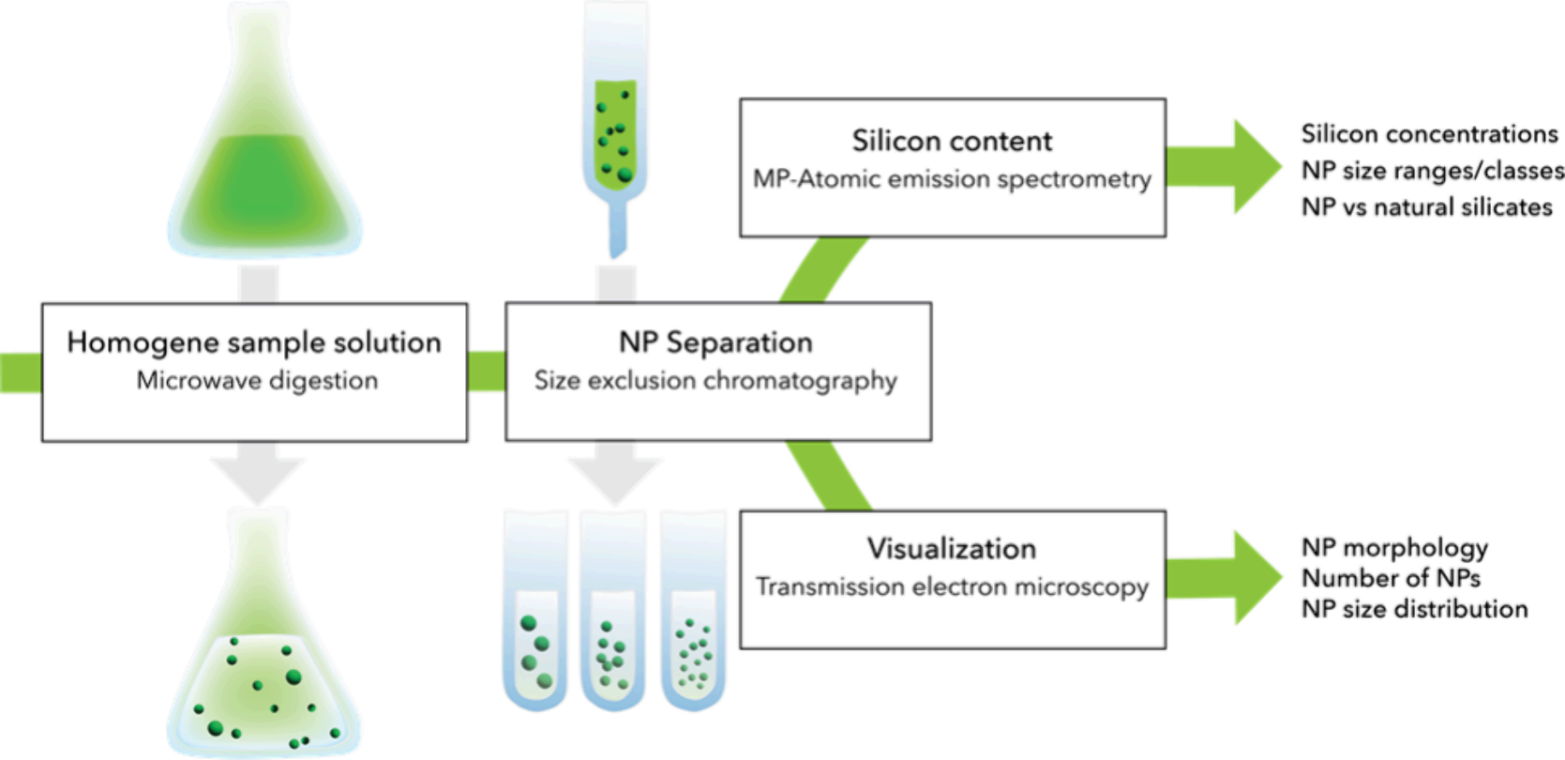
Suggested characterization approach for quality control of products containing SiNPs, offering a high-throughput solution for the swift assessment of multiple production samples. It will start with a digestion process, followed by a separation via size exclusion chromatography and finally an analysis by microwave plasma atomic spectroscopy (MP-AES). It quantifies a range of NPs, for example 30 μg of SiNP with a diameter range of 20–100 nm in a 1 L sample. Elaborative imaging with a transmission electron microscope and automated image processing enables the clear assessment and characterization of nanoparticles with regard to size, size distribution, morphology, agglomeration, etc. This method not only helps to estimate a NP contamination but also enables quantifying and defining lower limits and diameter range for so-called NP-free products.

Although characterization methods of pure nanomaterials are well established, they are not yet suitable for a detection of NPs in a complex matrix usually found in consumer goods, e.g. lotions, creams, biological fluids, foods, etc. Depending on their material properties, they are difficult to isolate and visualize. Matrixes not only interfere with measurements but can also lead to coating, encapsulation, aggregation, etc. According to Gottschalk et al.,^11^ an accurate detection and quantification of engineered particles requires their separation from natural particles and dissolved materials. Though, because SiNPs are chemically inert, are not dense, nor have specific properties as magnetic or metal particles, a distinct detection is difficult, as many described methods are not applicable. Thus, only few methods have been published, e.g. the characterization of SiNP in bentonite clay^12^ or in vitro and artificial digestions of foods containing silica as a food additive.^13^ The recovery range of SiO_2_ added to the matrixes ranged widely between 59 and 128% even when various online in process monitoring techniques such as nanoparticle tracking analysis (NTA), multiangle light scattering (MALS), and inductively coupled plasma mass spectrometry (ICP-MS) were applied.^14^ Characterization of SiNPs is an exceeding challenge because nutrition products often contain natural nonparticulate silicates. In addition, the composition of nutritional products can significantly differ, encompassing a wide range of ingredients and varying concentration levels. Adequate sample preparation methods that either cover all matrices or at least preparation protocols for each of the matrices are missing. A complex scenario in determining the amount of SiNPs involves evaluations at low concentrations, the coexistence of natural silicate, the property of SiNPs to form aggregates (at pH = 2–3),^13^ and the potential signal interferences from sample matrices. All these factors can lead to false positive or negative results, affecting the measurement accuracy, or alter the behavior of SiNPs. To overcome these challenges, it is important to consider a defined sample preparation step, distinguish characterization methods, optimize experimental conditions, and employ suitable reference materials for the characterization and analysis of SiNPs. Therefore, quantitative information about the amount or concentration of NPs has the highest priority. Furthermore, qualitative information, e.g. size, shape, state of dissolution, and aggregation, can be of great importance to study the effect of the NP in more detail. In addition, the quality control of multiple parallel production lines is expected to result in an increasing number of collected samples. Therefore, sample preparation and characterization methods have to be fast and provide clear information. Unfortunately, automated processes in NP characterization techniques are rare and often elaborate. Imaging techniques such as SEM, STEM, and TEM are often employed, in accordance with EFSA’s recommendations. However, a changing matrix is a challenge for automation. Automated imaging analysis in high-throughput microscopy (HTM) requires appropriate algorithms and well-prepared samples. Here we aimed to establish a quantitative characterization method (Figure 1) for nanoparticle contamination in complex matrices (e.g., early life nutrition, ELN) with the highest possible accuracy and automation level.

The findings are categorized into four sections: (A) investigating ELN-sample homogenization using three distinct digestion methods based on reactive oxygen species, dissolution, and acidic oxidation (Fenton, DMF, and microwave digestion) along with total silicon content measurement, (B) advancing the NP-separation method utilizing size exclusion chromatography (SEC) and utilizing SiNP standard references of varying diameters (20, 50, and 80 nm), (C) merging the separation and quantification of ELN samples with a complex matrix (as illustrated in Figure 1), and (D) characterizing with transmission electron microscopy (TEM) and image processing with Matlab.

## Experimental Section

### Samples

Noncommercial premix samples, Form 1 and Form 2 with and without silica AA and with different compositions, were provided by DSM Firmenich AG (Switzerland) as a test system. PBS-Tablets from Calbiochem were used containing 140 mM NaCl, 10 mM phosphate buffer, and 3 mM KCl, at pH 7.4 and 25 °C.

### SiNP and Silicon Standards

Standard SiNP powder with defined diameters (ϕ) of 20, 40, and 120 nm were purchased from General Engineering and Research LCC (USA). 80, 50, and 20 nm diameter SiNP dispersions were obtained from NanoComposix Europe and together with a certified ammonium fluorosilicate ((NH_4_)_2_SiF_6_) standard solution for atomic absorption spectrometry (AAS) from Carl-Roth AG the solutions were used to compare the different particles and methods under reduced complexity and for validation.

### Sample Digestion Methods

#### Fenton Digestion

A Radleys Carousel 12 Plus instrument (Radleys, UK) equipped with 20 mL tubes was used to run all samples in parallel under the same conditions. First a 100 mg sample was diluted in 750 μL of MQ-water. Then 50 μL of 0.1 M FeSO_4_·7H_2_O and 200 μL of hydrogen peroxide (35%) were added to the mixture. A magnetic stirrer was used at 700 rpm, and the samples were heated to 60 °C overnight. The solution turned a turbid yellow. 200 μL of NH_3_OH (2.4%, aqueous) was added to the solution and stirred at RT. Sample mixtures turned clear.

#### DMF Digestion

A Radleys Carousel 12 Plus instrument (Radleys, UK) equipped with 20 mL tubes was used to run all samples in parallel under the same conditions. A 500 mg sample was diluted in 2 mL of *N*,*N*-dimethylformamide (Merck, >99%) and stirred until a clear solution was obtained. Then 8 mL of HNO_3_ solution (1%, aqueous) was added and stirred overnight at 800 rpm.

#### Microwave Digestion

Samples were prepared in digestion vessels (20 mL) of a Mars 6 microwave (CEM Cooperation, USA). All ELN samples were run in parallel under the same conditions. A 500 mg sample was diluted with 4 mL of HNO_3_ (65%). The “Organic Method” was applied that heats the samples to 200 °C. Vessels were carefully opened (*Caution*! nitrous gases and pressure!), and samples were transferred into glass vials. Then samples were neutralized by adding 1 mL of concentrated NaOH or 1 mL of NaOH (1 mol/L) (DSM Form 1) to bring the pH to >3, and MQ-water was added to bring the total volume to 2 mL. Concentrations were calculated.

### Size Exclusion Chromatography

An Aekta Purifier FPLC instrument with conductivity and pH cell, 200 μL sample loop, and fractionator (for 10 mL centrifugation tubes) was used for automated size exclusion. Two types of columns were used. (A) A commercially available Superose 6 Increase 10/300 GL column (Mw 5k-5M, Cytiva Europe GmbH) was used with PBS buffer (PBS tablet, Calbiochem) with 0.05% SDS (Merck) as eluent. Washing was done with one column volume (CV) of 0.5 M NaOH solution, the maximum pressure was 1.5 MPa, and a flow rate of 0.5 mL/min was used. (B) A Sepharose4B column was prepared with Sepharose4B (Sigma-Aldrich) in a XK column (10 × 200 mm) with a final bed height of 120 mm; the maximum pressure was 0.3 MPa with a flow rate of 0.3 mL/min, and PBS with 0.05%SDS was used as an eluent. The column was regularly washed with 0.5 column volume (CV) PBS, 1CV MQ-water, 1CV NaOH 0.5 M, 1CV MQ-water, and 0.7CV PBS, to remove residual particles or matrix.

### Dynamic Light Scattering

The size distribution of samples was measured with a Zetasizer NS instrument (Malvern Instruments Ltd.) in 0.5 mL disposable PMMA cuvettes. The refractive index (RI) of the material was defined as 1.49 and material absorption as 100. The dispersant was water with a dispersant RI of 1.33 and viscosity of 0.8867. Each sample was measured three times. The method was used to measure dry standard SiNPs with diameters of 40–120 nm and diluted in MQ-water.

### Analysis with MP-AES

An Agilent 4210 MP-AES instrument equipped with an SPS 4 autosampler for 10 mL centrifugation tubes was used to measure the microwave generated emission peak of silicon at 251.611 nm. All solutions were diluted with 1 mL of matrix (0.1% nitric acid) to a minimum volume of 1.75 mL. An AAS silicon standard solution from Carl Roth GmbH + Co. KG (Art. No. 2350.1) was used to prepare five standard solutions (concentrations: 0.1, 0.5, 1, 5, and 10 mg/L) in 0.1% HNO_3_. Each sample was measured three times, with pump speed of 15 rpm, stabilization time of 10 s, and nebulizer flow rate of 0.5 L/min. Tubing and nebulizer were rinsed with MQ water after each measurement.

### Analysis with ICP-MS

Characterization of solutions of digested samples was carried out with an ICP-MS Agilent 7800 instrument. AAS Si standards with concentrations of 0–500 ppb were run with each measurement. Samples were diluted with MQ-water aiming at a Si concentration of ∼1 μg/L in order to fit into the standard curve.

### Transmission Electron Microscopy

Four microliters of a sample solution (SEC fractions or digested samples) was placed on a 200 mesh copper grid, carbon-coated Formvar (Electron Microscopy Science CF200-CU-50), and dried for 1 h. Buffer salts were then removed by adding 2 × 4 μL of MQ-water and removing it with a dust-free tissue (Kimwipes). Samples were then dried overnight and finally measured with a Zeiss EM300 microscope equipped with an AMT-XR280S camera at 50 kV.

### X-ray Photoelectron Spectrometry (XPS)

Titanium foil pieces of approximately 4 × 7 mm^2^ were cleaned with UV/ozone for 10 min. Immediately afterward a 10 μL drop of each digested sample was dried on the foil overnight. Samples were then loaded into the XPS vacuum prechamber of the PHI 5800 system and again evacuated overnight. After transfer into the main chamber (approximately 5^–10^ Torr) samples were measured with an Mg Twin Anode at 10 kV and 10 mA. Survey spectra were taken at a pass energy of 187 eV and 3 sweeps and detail spectra of C 1s, O 1s, N 1s, and Si 2p regions at a pass energy of 23 eV, 8–12 sweeps and 0.05 eV/step. Spectra were evaluated with CasaXPS software. Regions of interest were defined manually, and peak areas were measured using a Shirley Background and a Gaussian–Lorenzian curve fitting (GL30). In each nitrogen detail spectrum, peaks of the different nitrogen species were fitted with equal full-width at half-maximum (fwhm) values.

## Results and Discussion

Six powder samples of ELN formulations were provided by DSM Firmenich AG to test the method proposed. Three samples were known to contain the silicon-based anticaking agent (AA) Tixosil (Solvay SA), whereas the other three were supposed to be AA-free. They were collected at either different processing steps (Premix) or from different starting materials (Forms 1 and 2) (Table 1).

**Table 1:** Six Early Life Nutrition Samples Collected at Different Processing Steps Provided by DSM Firmenich AG

premix without AA	Form 1 without AA	Form 2 without AA
premix with AA	Form 1 with AA	Form 2 with AA

### Sample Preparation and Digestion Methods

Pure, amorphous Tixosil powder was used as reference; however, it formed agglomerates (Figure SI-1) when dispersed in MQ water and neither the addition of surfactants, e.g., Tween20 or SPAN85, nor the use of organic solvents, e.g. methanol, ethanol, ethyl acetate, toluene, led to an acceptable dispersion. In food processing, AAs are usually spray coated onto the food powder, which leads to a better distribution and, when dissolved, to a better dispersion. Therefore, we decided to use SiNPs of different diameters, both in solid form (ϕ 20, 40, 120 nm) and as a dispersion (ϕ 20, 50, 80 nm), as reference materials.

Quantitative characterization requires powders to be homogeneous; NPs must be accessible and dispersed. The provided Premix and Form 1 and 2 samples contained matrices full of various natural polymers, such as starches, sugars, etc., which were not soluble in aqueous solutions; they formed yellow-white emulsions. Despite the fact that ethyl acetate and the addition of surfactants (Span85) enhanced the solubility, the surfactants formed vesicles and the remaining organic part led to a thick opaque coating that disturbed both analytical methods, imaging by transmission electron microscope (TEM) (Figure SI-2) and size measurement with dynamic light scattering (DLS). For artifact-free samples and as proposed by Gottschalk et al.,^11^ the matrix needs to be removed, without removing or modifying the SiNPs present in the solutions.

**Figure 2:**
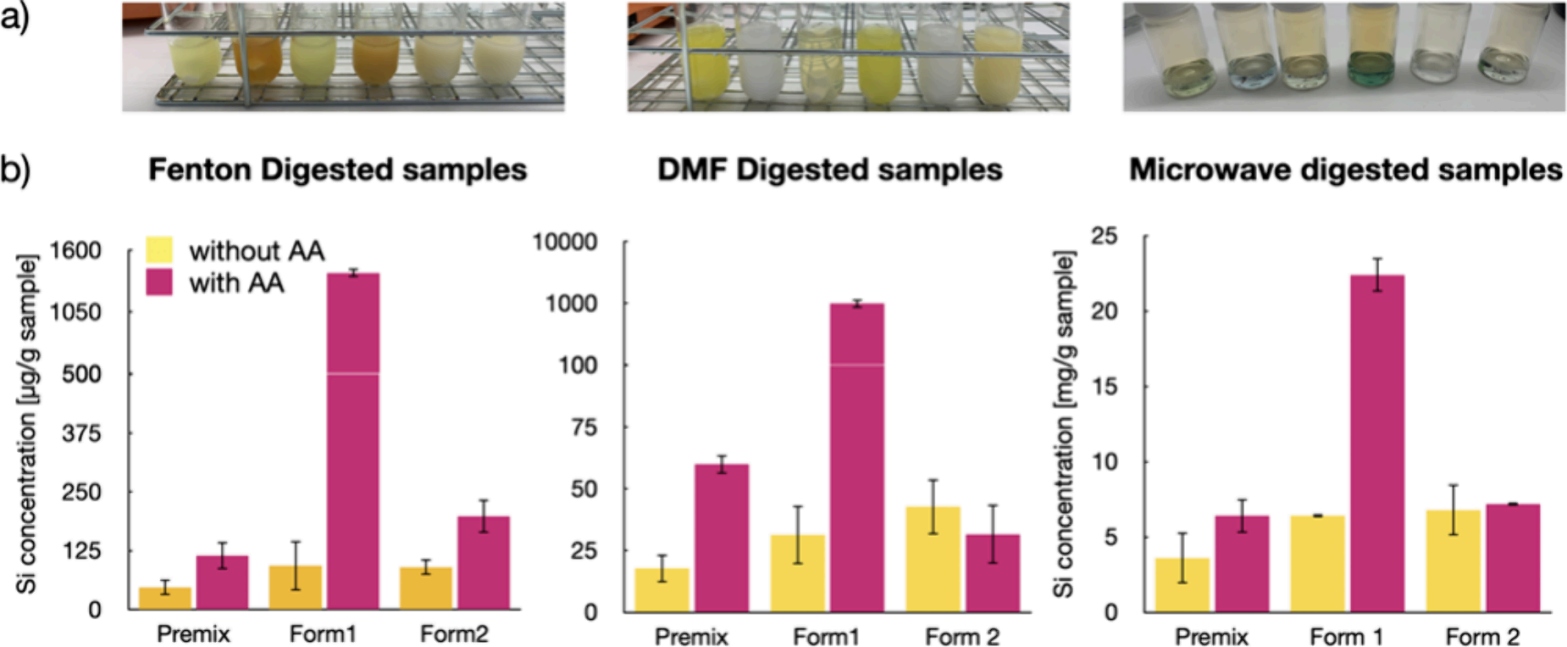
(a) Photographs of six ELN formulations (order of samples in the respective images from left to right: Premix without AA, Form 1 without AA, Form 2 without AA, Premix with AA, Form 1 with AA, Form 2 with AA), after different digestion methods: Fenton reagent, DMF/nitric acid, and microwave digestion. (b) MP-AES measurement of the total silicon content in each of the six digested ELN samples with and without AA. Overall higher values were measured for the microwave digestions. Thus, the *y*-axis scale was adjusted from a μg/g sample (Fenton and DMF) to a mg/g sample (microwave digestion).

Therefore, the organic matrix was reduced by three digestion methods: (a) Fenton digestion,^15^ (b) DMF digestion according to Azcarate et al.,^16^ and (c) digestion by a MARS 6 microwave.^17^ The two first digestions were performed in a Radley carousel to treat all six samples in parallel. In general, 500 mg of each sample was used. While the Fenton and the faster DMF digestions still led to turbid samples and sometimes formed two layers, the microwave digestion led to clear solutions (Figure 2a). Because the harsh conditions of this digestion are known to change particle morphology and lead to agglomerations,^18^ we used nitric acid solely and sample concentrations (125 mg ELN sample/mL HNO_3_ (65%)) as high as possible. According to the TEM images, the standard SiNPs (20, 50, and 80 nm) remain colloidal after the microwave treatment (Figure SI-17). However, the colloid surface shows some increased roughness best visible for the smaller particles. All three digestion methods enabled the imaging by TEM and the quantitative measurement of total Si with microwave plasma atomic spectroscopy (MP-AES) (Figure 2b).

### Measurement of Total Silicon Content in ELN Samples

The total silicon contents of the six digested ELN samples were measured with MP-AES (Agilent 4210 MP AES), because of the following reasons. (a) The sensitivity of MP-AES was sufficient for the Si content in the measured samples. Results were comparable to inductively coupled plasma mass spectrometry (ICP-MS) (Figure SI-3). (b) The handling of the instrument was straightforward. (c) The autosampler allowed for the rapid measurement of numerous (<192 in our setup) samples. ICP-MS single-particle analysis enabled differentiating between natural nonparticular silicates and SiNPs. We have performed some preliminary measurements with 20, 40, and 120 nm SiNPs and unfortunately did not find a significant difference for the 40 and 20 nm SiNPs (Figure SI-16). This agrees with the literature, where it applies solely upon NP diameters of >50 nm and therefore does not apply for the AA that was found to be approximately 20 nm.^19^ Therefore, we focused on the MP-AES.

#### Microwave Plasma Atomic Spectroscopy (MP-AES)

The quantitative measurement required the comparison of dispersed SiNP with free dissolved Si. Therefore, silica concentration of the different SiNP references (ϕ 20, 50, and 80 nm, 0.1–10 mg/mL SiO_2_ in H_2_O) were measured against a certified ammonium fluorosilicate ((NH_4_)_2_SiF_6_) standard solution (Si-AAS standard, Carl Roth AG) (Figure SI-4b). The standard curves of the three SiNPs (ϕ 20, 50, and 80 nm) are linear and the same for all diameters. However, the sensitivity of SiNPs is approximately 10% of the free Si-AAS standard curve (Figure SI-4a). A lower value is expected, as the Si of the NPs is not dissolved and therefore only the surface Si atoms are measured. The concentrations measured in the following graphs still refer to the Si-AAS standard, unless otherwise stated.

The total silicone content was then measured with MP-AES for the three digestion methods and the six ELN samples. Although the values showed a similar trend (Figure 2b), the absolute values varied a lot depending on digestion method: for example, for the premix without AA from 25 μg to 3 mg/g sample. Microwave digested samples showed the highest measured Si concentrations ranging from 3 mg/g Premix without AA to 23 mg/g Form 1 with AA, which corresponds to 0.3% and 2.3%. This is in agreement with the expected <4% AA levels in products. We observed a foam-phase separation in the Fenton and DMF digested samples and suspect that some AA remained in the foam phase, resulting in lower Si concentrations compared to the clear microwave solutions and probably also to higher standard deviations as well. Correspondingly, the microwave digestion was used for further method development.

Silicon was found in all the samples, including the three “AA-free” ELN samples. This discovery is not particularly unexpected, as ELN samples frequently contain natural silicates in a nonparticulate state. Moreover, the difference of total Si concentrations between with and without AA was only significant for Form 1 and not for the premix (Figure 2b). Form 2 behaved unexpectedly; it seemed like there was more Si in Form 2 without AA than in Form 2 with. Indeed, Form 2 did not pass the DSM product specification and the reason might be the low AA concentration. For future method development, we concentrated mainly on the Premix/Form 1 samples.

#### X-ray Photoelectron Spectroscopy (XPS)

Further approaches to measure the relative silicon content of the Fenton reagent digested premix samples were done by XPS, where a drop of each digested solution was dried onto a titanium foil substrate and measured under ultrahigh vacuum. XPS gives the relative atomic percentage of elements in the samples such as Si, which is measured via Si 2p peak. The Si atom % was significantly higher for the Premix sample with AA with respect to that without (Figure SI-5). However, the method does not allow for absolute quantification of silica.

The most effective method for quantifying total silica content was determined to be MP-AES. However, in order to differentiate between particles and nonparticles, minimize image artifacts, or specify diameter ranges, it is necessary to combine this technique with a separation method that can eliminate the matrix and even provide resolution of particle sizes, such as size exclusion chromatography (SEC).

### Separation with Size Exclusion Chromatography (SEC)

The combination of SEC and silicon content measurements by MP-AES was first evaluated with pure SiNP reference particles of different diameters. Depending on the column, SEC could also separate NPs with different diameters and thus allow classifying the NPs: e.g., >250 or <250 nm. Therefore, an Aekta Purifier was used and equipped with two different columns to compare the efficiency, a commercially available Superose 6 GL30/100 column and a self-packed Sepharose 4B column. Methods were developed using SiNP references of three diameters: 20, 50, and 80 nm. Collected fractions were then diluted 1:1 with nitric acid (0.1%) and the silicon concentration directly measured with MP-AES.

The Superose 6 GL30/100 column showed a distinct concentration dependence for the Si peak after an elution time of 42 min for all types of SiNPs. Unfortunately, the column did not separate the different sized SiNP references (20–80 nm) (Figures SI-6–SI-8)

**Figure 3:**
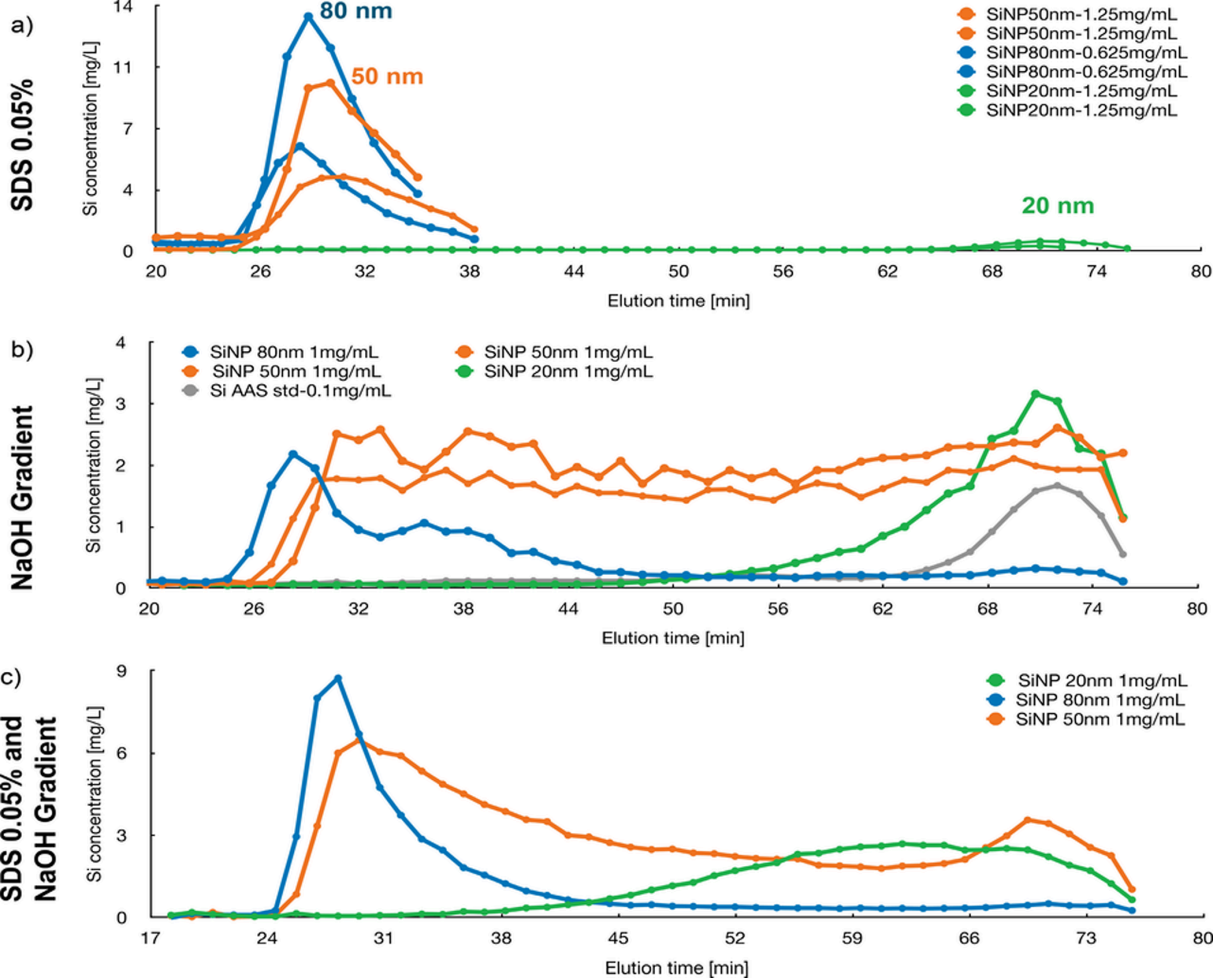
Influence of eluent additives on size separation. 20 (green), 50 (orange), and 80 nm (blue) standard silica nanoparticles were separated on the same Sepharose 4 B column with PBS and (a) 0.05% SDS, (b) 20% NaOH (0.5 M) gradient, and (c) the combination of both.

The Sepharose 4B column matrix is known to separate nanoparticles^20^ and thus was chosen to run samples and standards (Figures SI-11 and SI-12). A method was developed to separate the three SiNP references, making it possible to subsequently apply the findings to the ELN samples. SiNP references and AAS silicone standard were each suspended in PBS and separated on a Sepharose 4B column with eluent (PBS: 140 mM NaCl, 10 mM phosphate buffer, 3 mM KCl, pH 7.4). The 0.05% SDS in the eluent allowed a separation of 50 and 80 nm SiNPs, but the 20 nm particles did not elute very well; they stuck to the matrix (the peak is small) (Figure 3a). In general, peak areas were not reproducible, and we observed cross contamination between the runs (Figure SI-18). Thus, we have compared the effect of 1% Tween20 and 1% NaOH in PBS eluent (Figure SI-19). Both are known to be compatible with Sepharose 4B and do not impair the column properties. To avoid the strong interaction of sample with the column, NaOH (0.5 M) was found to be most suitable. This adjustment resulted not only in the elution of 20 nm SiNP but also in peak broadening (Figure 3b). The combination of NaOH gradient and SDS (0.05%) finally led to better-resolved peaks in combination of higher SiNP elution (Figure 3c). The impact on peak separation and shape showed the importance of the appropriate eluent for separation, as already proposed by Wei et al.^21^

It has to be noted that the 20 nm particles overlapped with the added AAS Si standard, which was supposed to be dissolved ammonium fluorosilicate and expected to run at the end of the column (Figure 3b). Nevertheless, when measuring the dry AAS standard by TEM, similar NPs with diameters around 20 nm were found to be formed (Figure SI-9). This would explain why AAS Si standards elute simultaneously with the 20 nm SiNP reference. In order to determine the end of the column, a small fluorescent molecule (2-(4-pyridyl)-5-((4-(2-dimethylaminoethylaminocarbamoyl)methoxy)phenyl)oxazole) (PDMPO) was mixed into each sample by subsequently measuring fluorescence. The maximum peak height was found to be at 75.7 min (Figure SI-10) for the NaOH gradient method, which is 5 min later than the elution time for 20 nm particles.

### ELN Sample Digestion, SEC, and Measurement of Total Silicone by MP-AES

**Figure 4:**
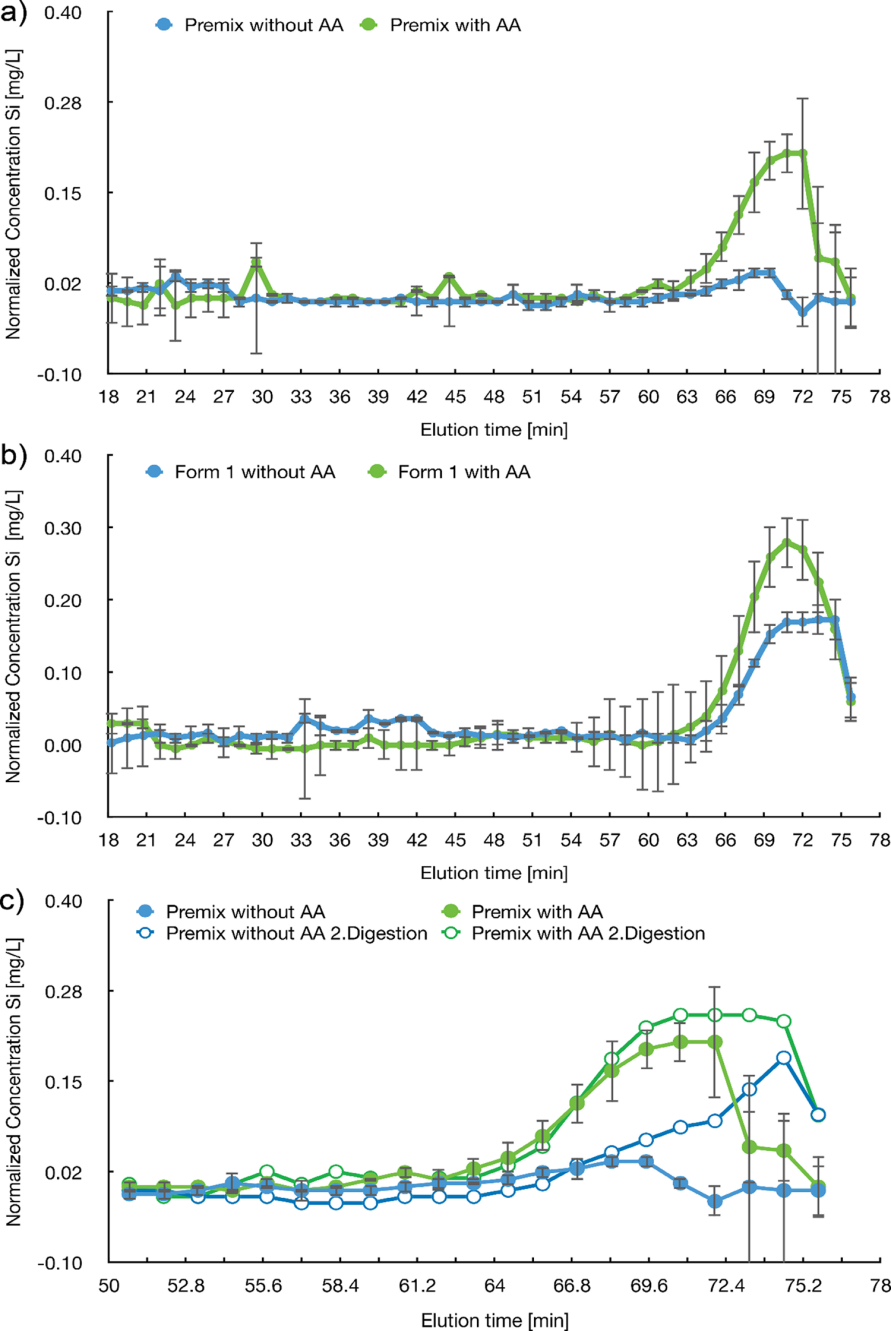
Silicon concentration in each size exclusion fraction of microwave-digested Premix (a) and Form 1 (b). The premix samples were digested in two batches (second digestion) and compared (c). SEC was done with an AEKTA Purifier system, equipped with a Sepharose 4B column and PBS with NaOH gradient (20% of 5 M solution). All fractions (duplicates) were diluted with 0.1% HNO_3_ solution (1.5:1) and measured with a microwave plasma atomic emission spectrometer.

These preliminary adjustments have facilitated the measurement of microwave-digested ELN samples in a sequential manner, yielding consistent and reproducible results (Figure 4). ELN samples were diluted (1:1 dilutions of digested sample in eluent (PBS and 0.05% SDS) equal to approximately 50 mg/mL). In comparison to the Superose 6 GL30/100 column (Figure SI-8), the overall Si concentrations measured for the Sepharose 4B column were higher with smaller standard deviations and therefore enabled resolving Premix with and without AA (Figure 4a). Also, Form 1 showed a significant difference between with and without AA (Figure 4b). Premix samples were also digested in duplicates by microwave and showed reproducible results (Figure 4c).

In summary, the combination of SEC and MP-AES applied on digested samples allowed distinguishing between Premix with and without AA with better sensitivity as measuring the total Si amount. It also showed a significant Si amount in Form 1 without AA, as already observed when measuring the total Si in the digested samples (Figure 2b). The elution time of 71 min showed that AA in the ELN samples has a diameter in the range of 20 nm, which is supported by TEM-imaging (Figure SI-13).

### Image Processing with Transmission Electron Microscopy by MatLab

Visualization of dissolved samples prior to digestion with transmission electron microscopy (TEM) was not possible, as the matrix dried on the sample and was not removed by washing with water. It formed oily films, with larger particles and salts, so that the particles were mostly covered or embedded by matrix residues and hardly isolated (Figure SI-2). The absence of contrast rendered automated image processing unfeasible.

**Figure 5:**
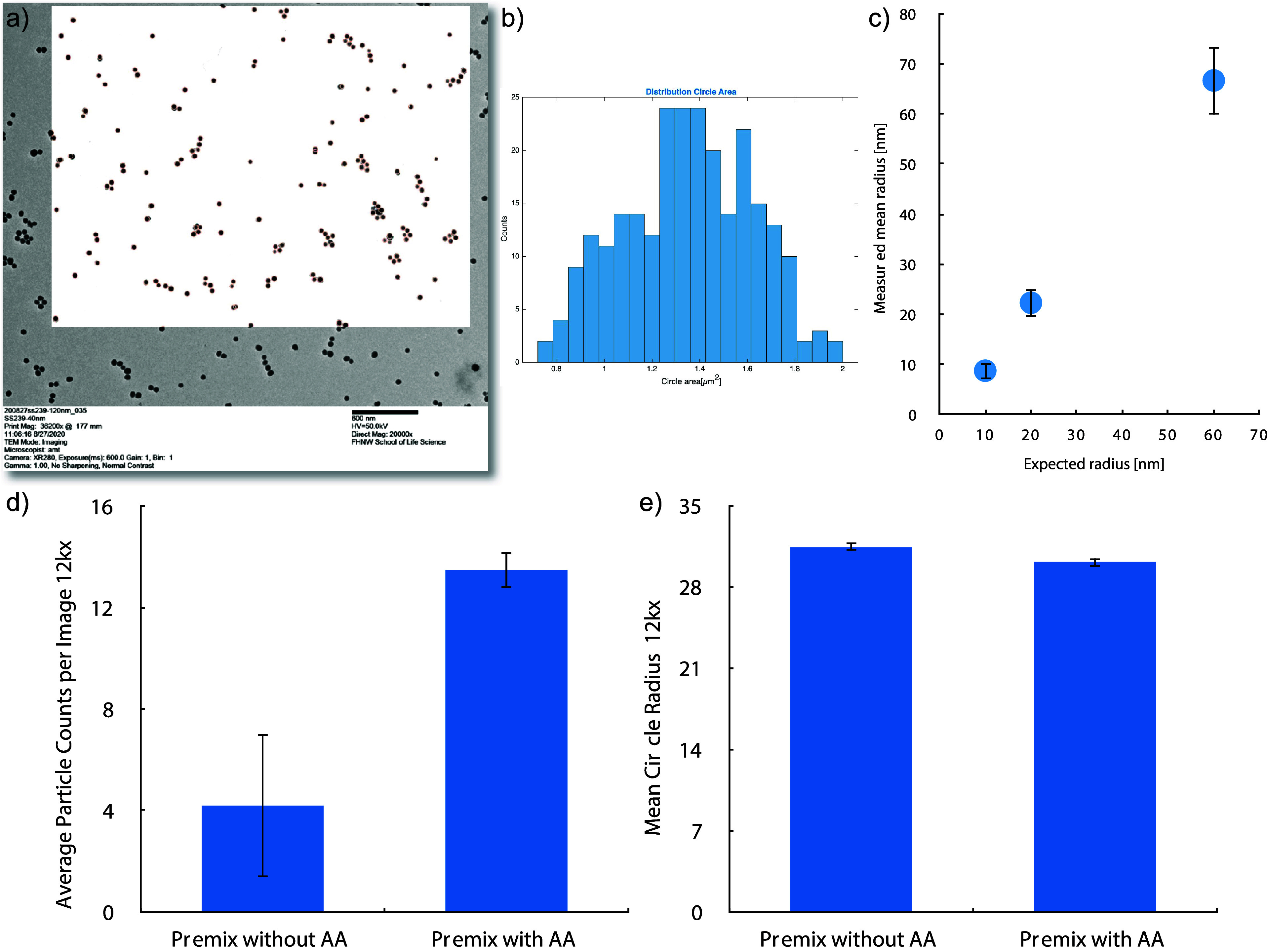
Image processing of standard SiNPs (powders ϕ 20, 40, and 120 nm). (a) TEM image of 40 nm SiNPs, with processed picture (white inset), where the red circles are generated by the findcircles function of MatLab from Matworks Inc. (b) Histogram of the circle area distribution found for the SiNP with 40 nm diameter. (c) Measured mean radius of the standard SiNPs after MatLab image processing of at least four images (50 kV, 20 kx and 50 kx for 20 nm). (d) Average particle counts and (e) average particle radius obtained from MatLab image processing on at least four transmission electron microscope images (50 kV, 12 kx) of each Premix sample with and without AA after Fenton digestion (duplicates).

However, the digestion step enabled at least the measurement of the particles. Nevertheless, the residual matrix continued to cause interference with the image, necessitating the implementation of washing steps to remove the matrix residue as much as possible. This approach also affected the particle concentration on the grid, resulting in the removal of more or fewer particles depending on the nature of the matrix. In addition, the acidic digestion solution causes degradation of the Formvar film of the TEM grids. A carbon-coated film was more stable, but the incubation time had to be less than 5 min. This allowed for image processing such as particle counting and size estimation using MatLab’s “findcircles” function (MatWorks Inc.). Initially, this function was tested on our SiNP standards (see Figure 5a) and then applied to TEM images of premix samples with AA and SiNP without AA subjected to Fenton digestion (see Figure 5b). Residues from the digested matrix are visible in the TEM images (gray residues, Figure SI-14). The black dots represent aggregates of SiNPs with an approximate diameter of 20 nm and are often surrounded by matrix material, resulting in their fuzzy appearance. Particle counting was carried out using MatLab on two distinct samples, each comprising at least two TEM images captured at a 12 kx magnification. The counts yielded in significant different results between Premix without AA (blue bars) and with AA (green bars) (Figure 5b). However, the large sample deviations highlight the need for method optimization to prevent counting of other residues and enhance the isolation of the particles.

Due to the difficulties in imaging caused by the residual matrix, the particles were finally isolated in different ratios by size exclusion chromatography (SEC). These SEC fractions finally allowed measurement by TEM. Despite the eluent (PBS buffer) still having to be removed, which likely led to some reduction in the number of NPs on the grid, it can be assumed that this reduction was consistent across all samples, as the matrix (eluent) remained constant. The TEM images of the fractions with the highest silicon concentrations of the SiNP standards show the correct particle sizes and comparable concentrations of particles per image (Figure SI-12).

**Figure 6:**
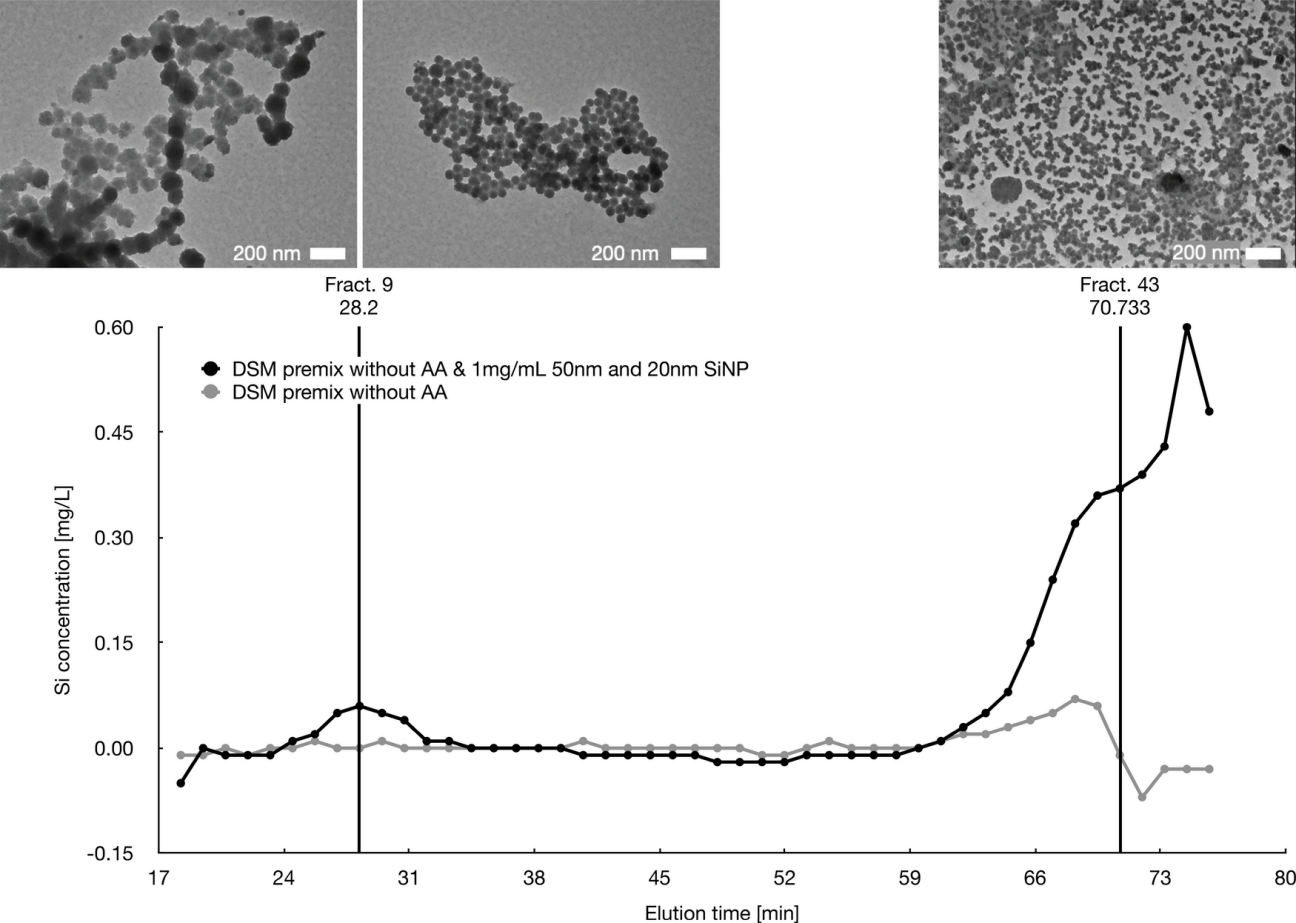
Size exclusion chromatogram of premix sample without AA spiked with 1 mg/mL 20 and 50 nm SiNP standard samples (black) and the premix sample without AA (gray). TEM images were taken from Fractions 9 (left and middle) and 43 (right), at 50 kx with a Zeiss EM900 instrument.

Moreover, a premix sample without AA was spiked with 1 mg/mL 20 and 50 nm SiNP standards, separated via SEC on Sepharose 4B column applying the NaOH gradient and SDS method (Figure SI-15). Fractions were then characterized by MP-AES and the ones with high Si content also by TEM. Both 20 and 50 nm particles were visible in TEM for eluents, but the 50 nm peak appears larger for the NaOH gradient and the 20 nm peak larger for the NaOH and SDS method, with TEM one can see that Fraction 9 contains also many 20 nm particles, that might agglomerate on the 50 nm particles. Whereas in the NaOH and SDS method particles seem to be more separated but no longer spherical, they appear disintegrated perhaps due to digestion or NaOH treatment. TEM images correlate with the enhanced separation by adding SDS to the eluent. The visualization of particles was important to understand and verify SEC data. The application of automated image processing on ELN samples gave a rough distribution and with a larger number of TEM images per sample the method would be promising for quantification (Figure 6). Nonetheless, the constrained TEM throughput and the absence of a multiplexing option substantially prolong the process, which is why it was not subjected to further assessment.

## Conclusion

The detection of nanoparticles in complex matrixes is a challenge, not only in terms of quantification but also in terms of shape and size definition. Within this project, we proposed a method for the fast detection and quantification of low concentrations (0.1–10 mg/mL) of SiNPs in complex matrixes, which can be utilized for quality control of nutritional products (Figure 1). The method effectively distinguishes nanoparticles larger than 20 nm from dissolved silicates and allows the quantification of nanoparticles within the size range of 20–120 nm. What sets this qualitative method apart is its capacity to quantify and determine size distribution in a sample, offering valuable insights into the shapes of individual particles and their agglomerates. The liquid handling of this method allows for full automation, with the current research protocol yielding results within 3.5 h. However, there is room for further optimization. Besides its utility with complex matrices, low detection limits, high throughput, and multisampling, this method is envisioned to be adaptable to other particle systems and matrices without necessitating equipment modifications, especially for particles smaller than 120 nm. During the project, we focused on high-throughput liquid sample handling; however sample preparation and size separation by SEC also enabled improving the quality of electron microscopy images. SEC effectively removed a significant portion of the interfering matrix and measured particle numbers in the same fraction increasing with increasing concentrations. Additionally, we recognize the potential for using TEM as a complementary technique to refine particle size distributions and quantification, while also providing insights into particle shapes and agglomerations. The implementation of multisampling techniques, similar to those used in microarrays in combination with automated image processing, would significantly increase sample throughput and enable measurement of each fraction within a single TEM analysis. Furthermore, the inclusion of an EDX detector could yield chemical information, supporting the quantification of nanoparticles.

In the future, we aim to expand this method’s applicability not only to SiNPs in diverse matrices such as creams, lotions, food products, and cell batches but also to other nanoparticle types (10–100 nm), including inorganic materials like TiO_2_ and CaP, as well as nanoplastics. This expansion will necessitate the development of new digestion protocols, different column matrices, and specific eluents, among other considerations.

## Supporting Information Available

Additional TEM images, SEC column setup, SEC and preliminary ICP-MS data, and MatLab script (PDF)
