# Supplementary material for: High-Throughput Silica Nanoparticle Detection for Quality Control of Complex Early Life Nutrition Food Matrices
Source: ACS Omega. 2024 Apr 9;9(16):17966–76. doi: 10.1021/acsomega.3c09459 (PMC11044250; doi:10.1021/acsomega.3c09459)
Supplement: Supplementary file 1 — ao3c09459_si_001.pdf [file ao3c09459_si_001.pdf]

Supporting Information

High-throughput silica nanoparticle detection for quality control of complex early life nutrition food matrices

Viviana Maffeis[1,2†], Andrea Otter[3], André Düsterloh[3], Lucy Kind[4], Cornelia Palivan[1,2], Sina S. Saxer[4*†]

[1]University of Basel, Department of Chemistry, Mattenstrasse 22, 4002 Basel BS, Switzerland

[2]NCCR-Molecular Systems Engineering, 4002 Basel, Switzerland

[3]DSM-Firmenich AG, Wurmisweg 576, 4313 Kaiseraugst AG, Switzerland

[4]FHNW School of Life Sciences, Institute of Chemistry and Bioanalytics, Hofackerstrasse 30, 4132 Muttenz BL, Switzerland

[*] Corresponding Authors

[†] V. M. and S. S. contributed equally to this study

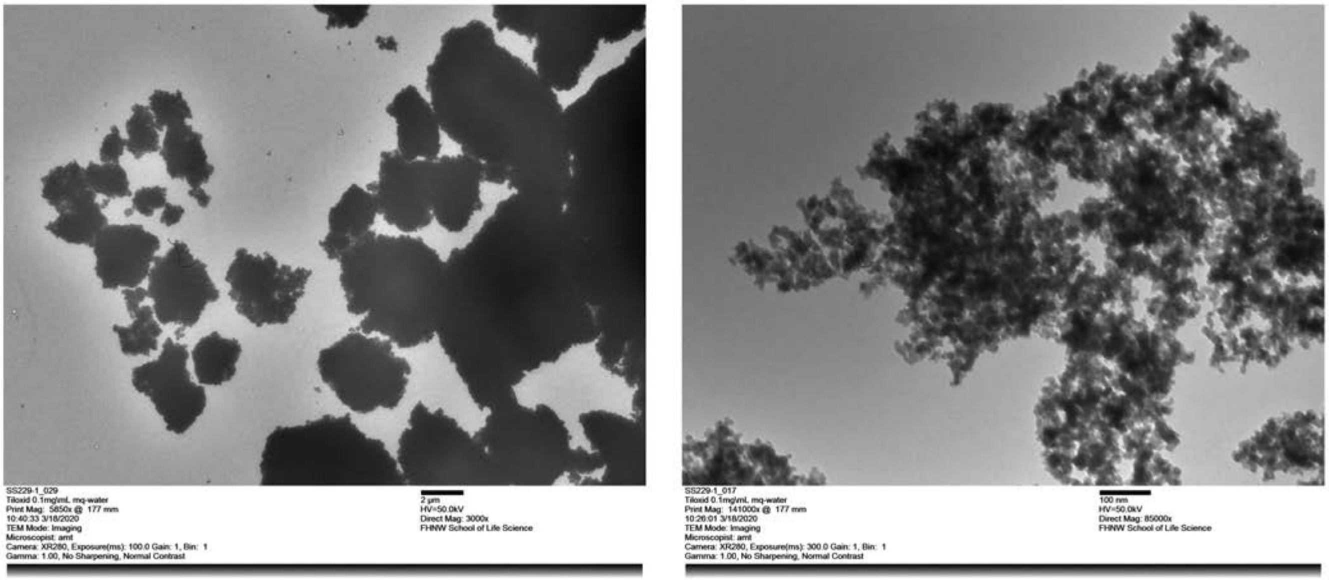

Figure SI-1. Transmission electron microscopy image of Tixosil 0.1mg/mL in MQ-water, dried on a Formvar coated copper grid. Measured with a ZEISS EM900, 50kV with a Magnification of 3000x (left) and 85000x (right).

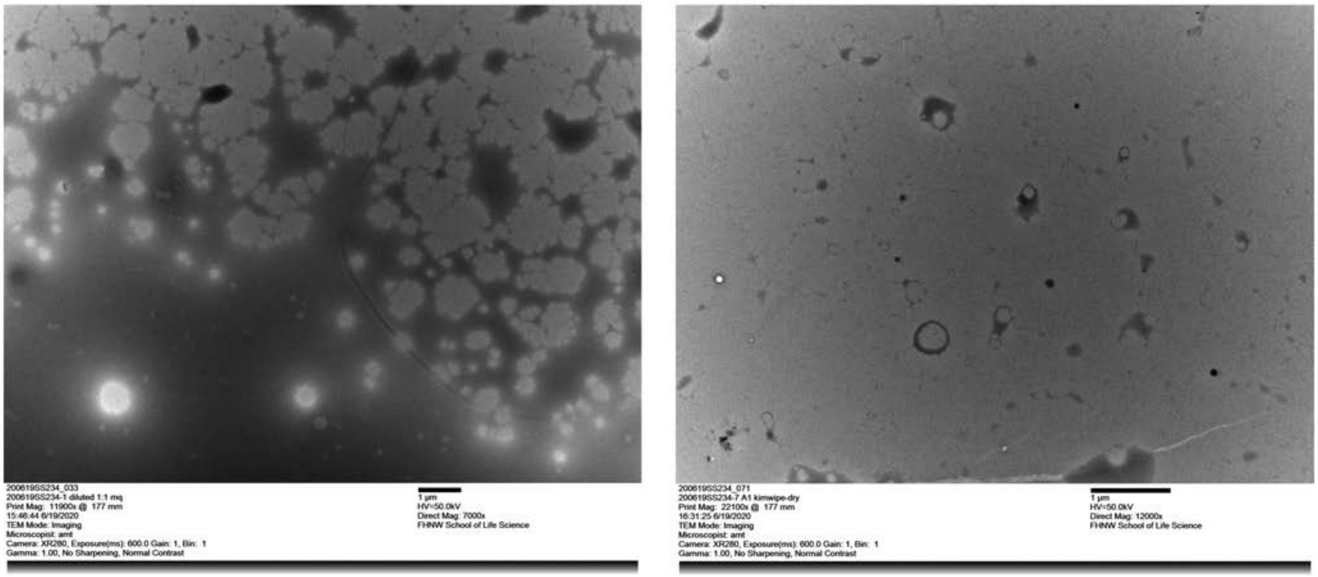

Figure SI-2. Transmission electron microscopy images of DSM Premix with and without AA (20 mg/mL in MQ water) taken on Formvar coated copper grids with a ZEISS EM900 at 50kV. Left image: 2µL of the 10x diluted premix with AA solution was dried on the grid and measured at 7kx. Right image: 2µl of Premix without AA solution incubated on the grid for 2 min until the drop was removed with a dust-free paper tissue. The image was taken at 12kx.

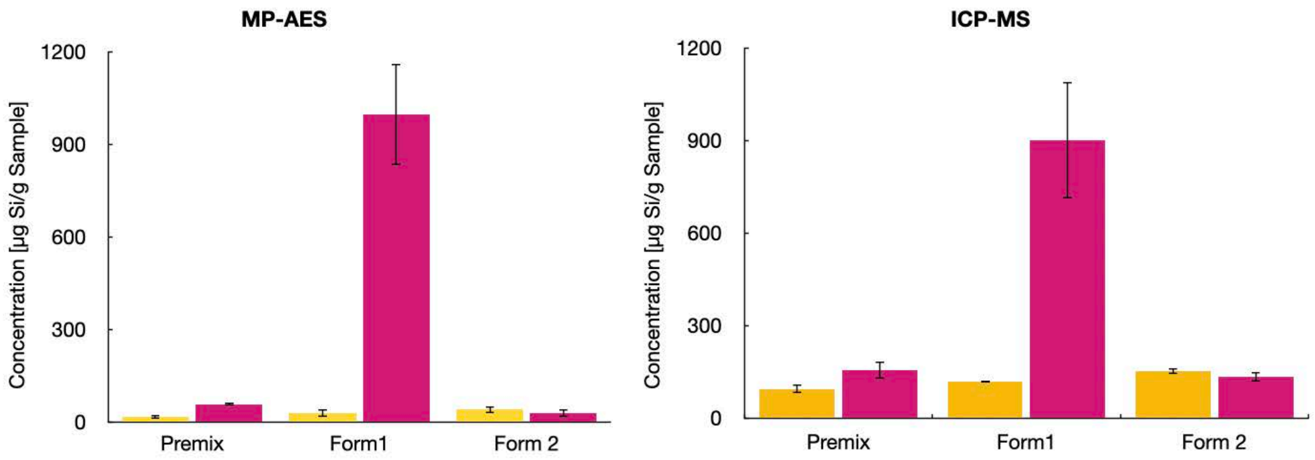

Figure SI-3. Total Silicon concentration of the DMF digested samples measured with MP-AES (right) and ICP-MS (left).

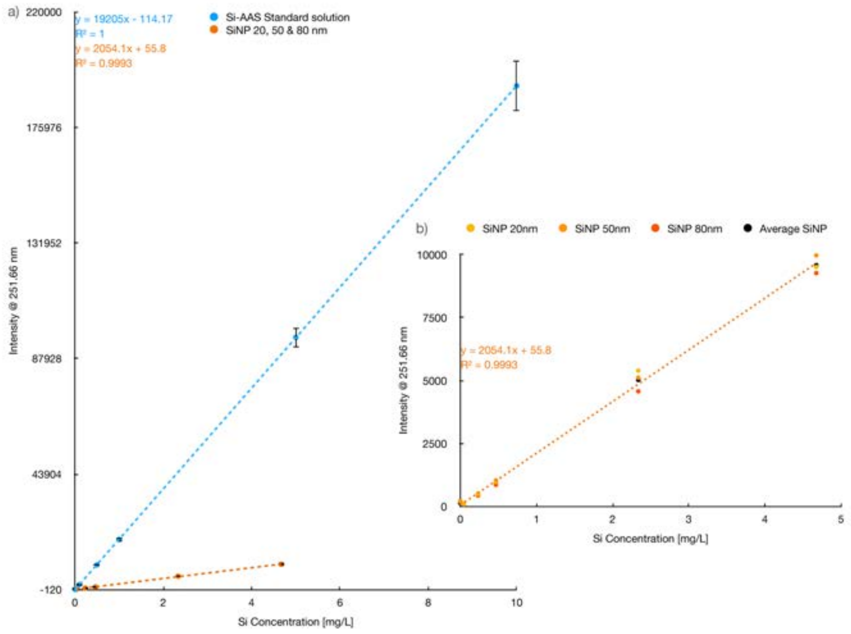

Figure SI-4. a) Comparison of intensity measured at 251.66 nm (silicon peak) of different concentrated silicon AAS standard solution (blue) versus SiNP standard solutions (red) measured by the MP-AES. Samples were diluted in 0.1% nitric acid solution. b) The resolved MP-AES intensities of the SiNP standard solutions with diameters of 20, 50 and 80 nm.

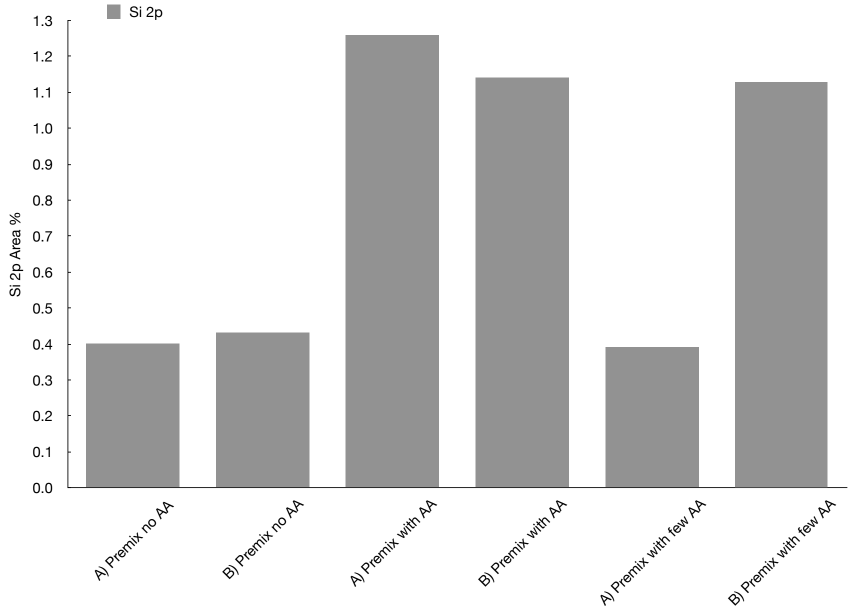

Figure SI-5. Atomic percentage (at%) of the Silicon 2p peak of Premix with and without AA and on Premix sample with only few AA measured by X-ray photo electron spectrometry. Powders were measured with a PHI 5800 spectrometer equipped with a Mg twin anode at 10kV & 10 mA, peak area were evaluated using CasaXPS. Each sample was measured twice A&B.

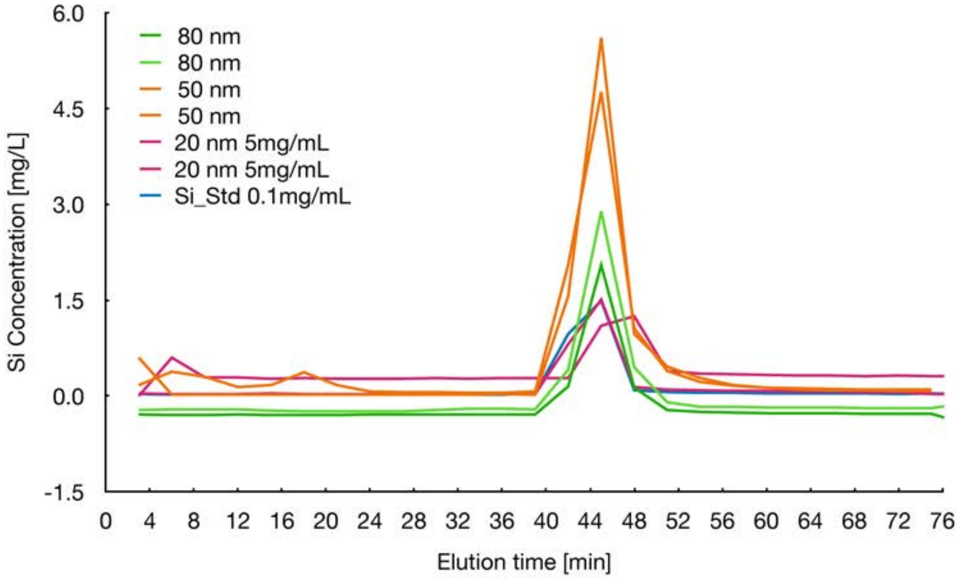

Figure SI-6. Silicon concentration in SEC fraction of SiNPs with different diameters (20, 50 and 80nm) and Si AAS Standard run on a Superose 6 GL30/100 column and analyzed by MP-AES. The column did not separate the different sized SiNP References.

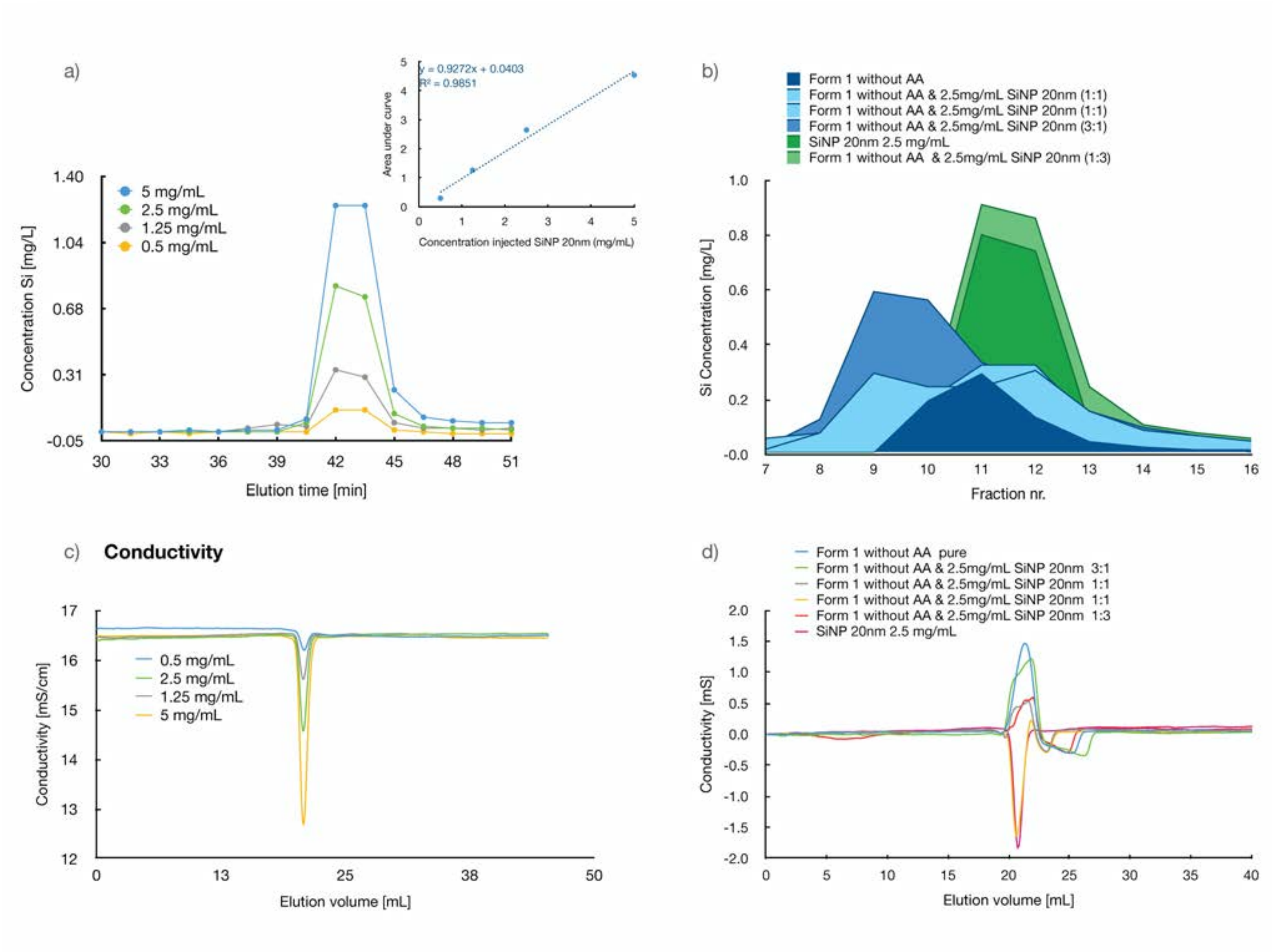

Figure SI-7 a) SEC of the SiNPs with Superose 6 column a) Silicon concentration of SEC fractions of 20nm SiNPs injected at concentrations of 0.5-5mg/mL. b) Spike experiment where DSM Formulation 1 without AA was spiked with different ratios of 20nm SiNPs (2.5mg/mL). Only a slight shift in elution time was observed. c) Conductivity measured in line with Aekta Purifier for the different concentrate 20nm SiNP (see a)), d) Conductivity measurement for the spike experiment (same as b))

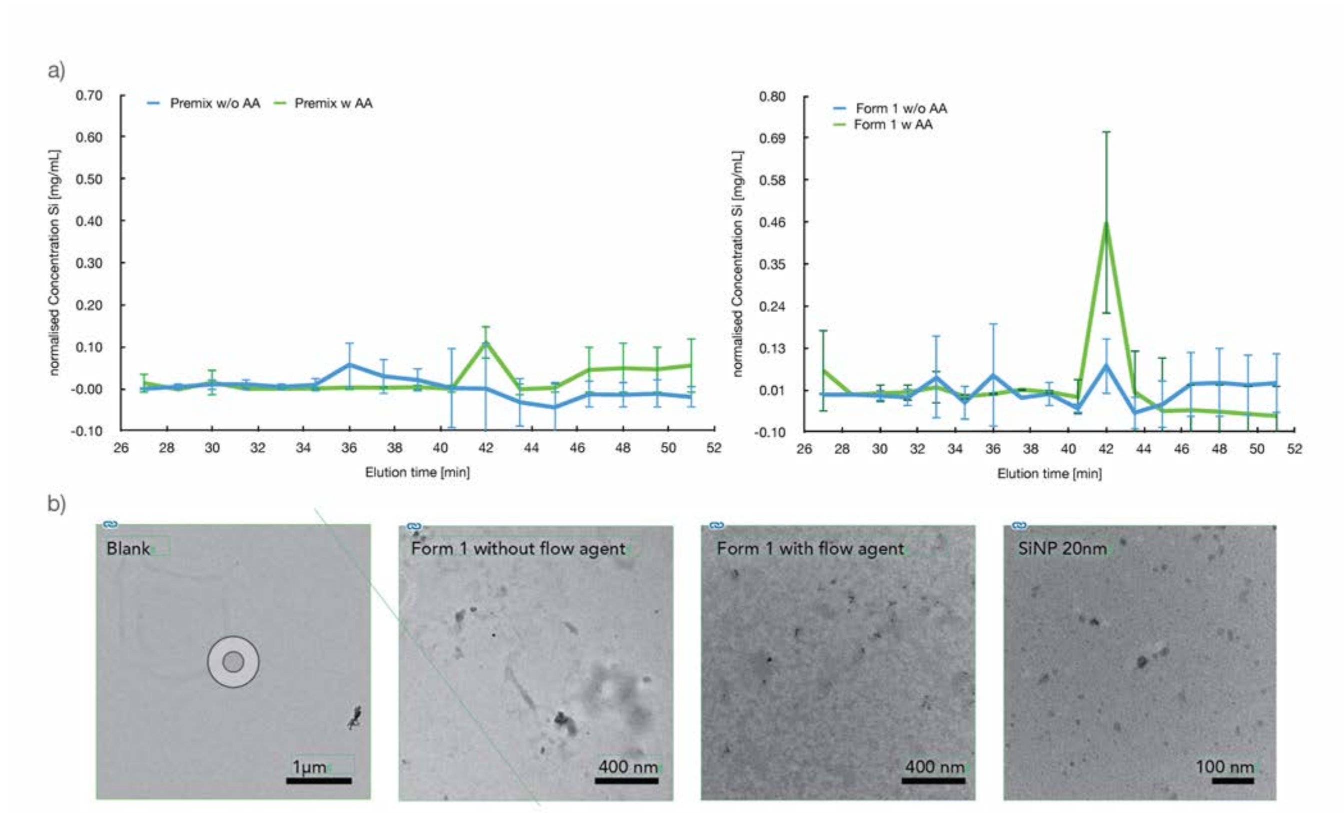

Figure SI-8: Si content in each SEC fraction of the superose 6 GL30/100. All fractions (triplicates) were diluted with 0.1% HNO_3 solution (1.5:1) and measured with microwave plasma atomic emission spectrometer with autosampler (Agilent 5200). b) Transmission electron microscopy picture of the SEC fraction #11 of a digested MQ water = blank, Form1 without and with flow agent and Standard SiNP20nm. 4 µL of all fractions were placed on a Carbon coated Formvar Cu grid, incubated and washed 2x with MQ water prior to the analyzation with a Zeiss EM900 microscope. Salt residues are still visible and both, Form 1 with and without flow agent.

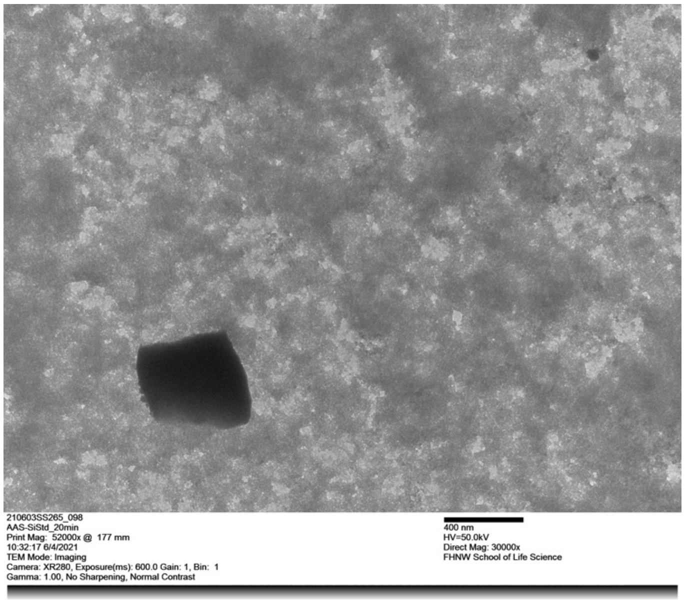

Figure SI-9: Transmission electron microscopy image of Si AAS standard ($(NH_4)_2SiF_6$Solution 1000mg/L) after SEC with Sepharose 4B column. The fraction of the Ammonium Fluorosilicate solution was dried on 200 Mesh, Copper Formvar TEM grid and washed twice with MQ water. The black square is a remaining salt crystal.

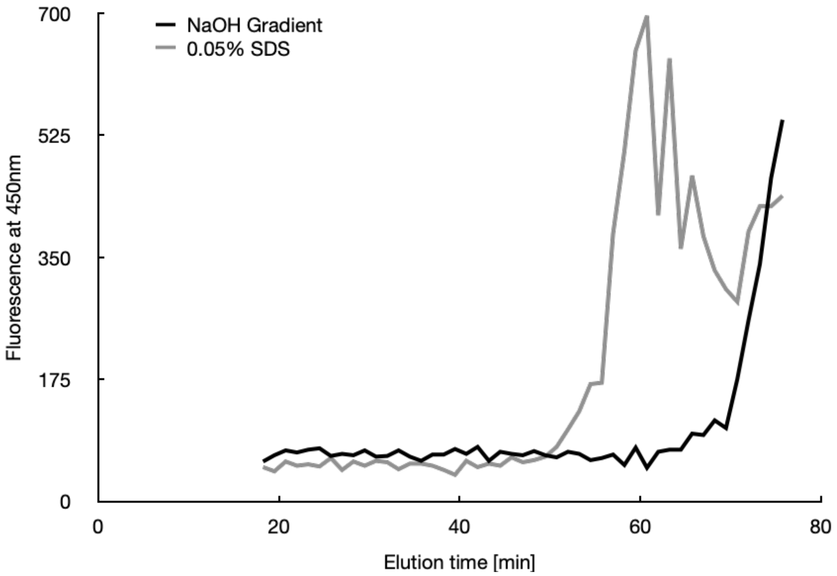

Figure SI-10 Size exclusion chromatography of the Fluorescence marker PDMPO on a Sepharose 4B column run with 0.05%SDS/PBS (grey) or NaOH 0.5M gradient method.

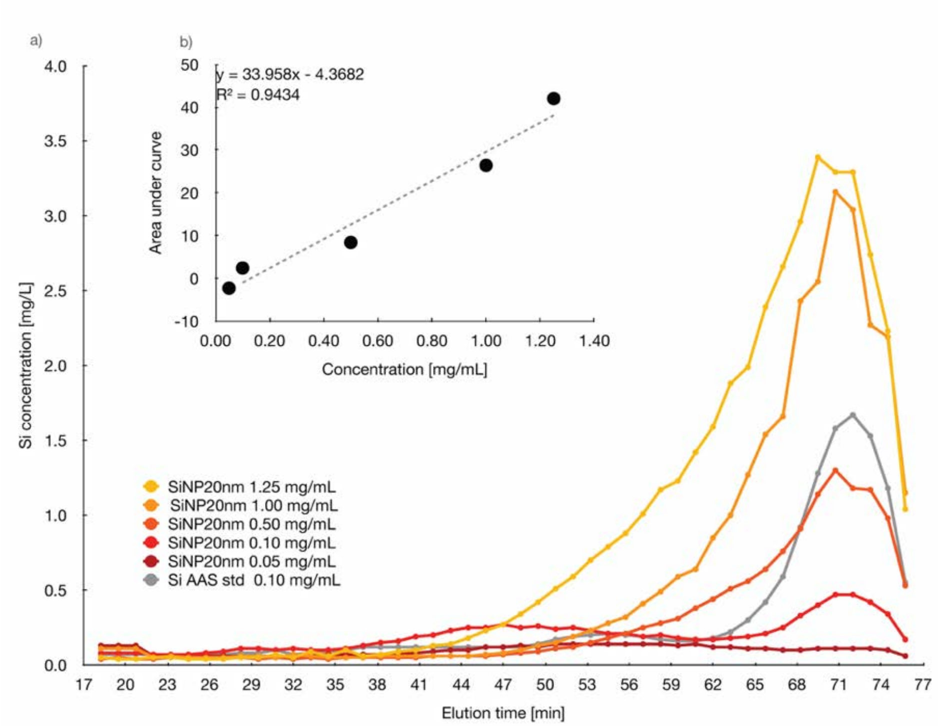

Figure SI-11 a) Silicon concentrations in SEC fractions of SiNP 20nm at concentrations from 0.05-1.25mg/mL (relish) and the Silicon AAS standard at a concentration of 0.1mg/mL (gray). b) Standard curve of SiNP 20nm area under the peak versus its theoretical concentrations.

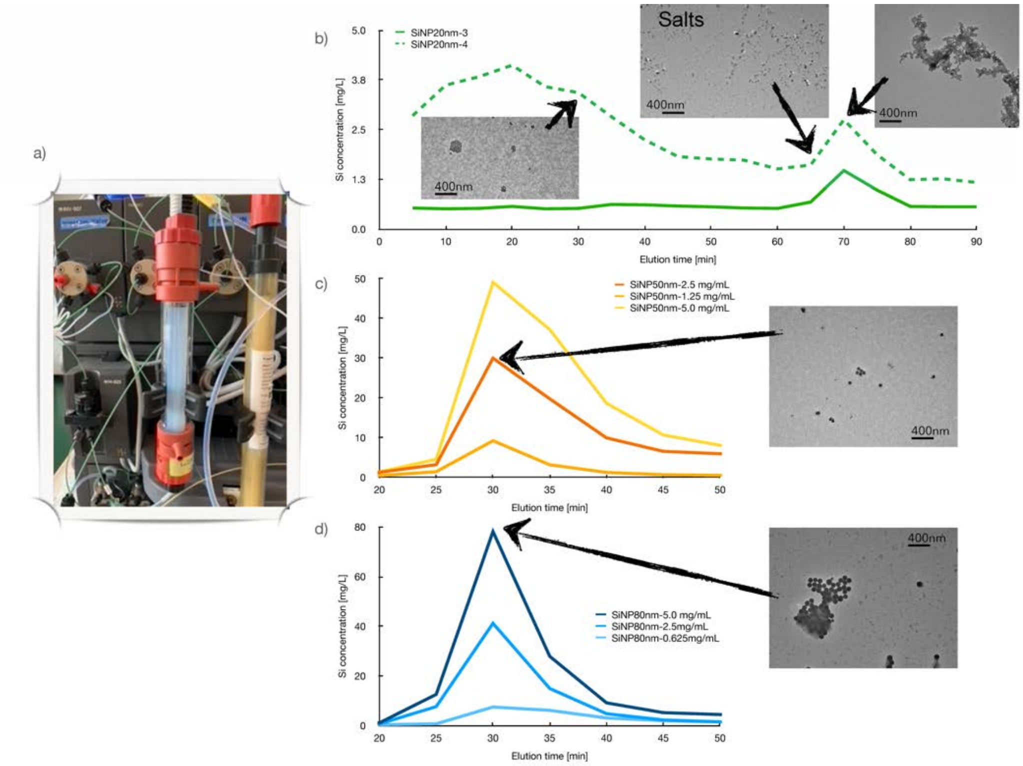

Figure SI-12: a) Photograph of Sepharose 4B Column (left) next to Superose 6 (GL30/100) column (right). b) Silicon concentration in SEC fractions of SiNP 20 nm 2.5mg/mL measured by MP-AES and transmission electron microscope images (at magnification of 30kx) of three fractions. c)Silicon concentration in SEC fractions of SiNP 50nm (1.25-5 mg/mL) measured by MP-AES and transmission electron microscope images (at magnification of 30kx). d) Silicon concentration in SEC fractions of SiNP 80nm (0.625-5mg/mL) measured by MP-AES and transmission electron microscope images (at magnification of 30kx).

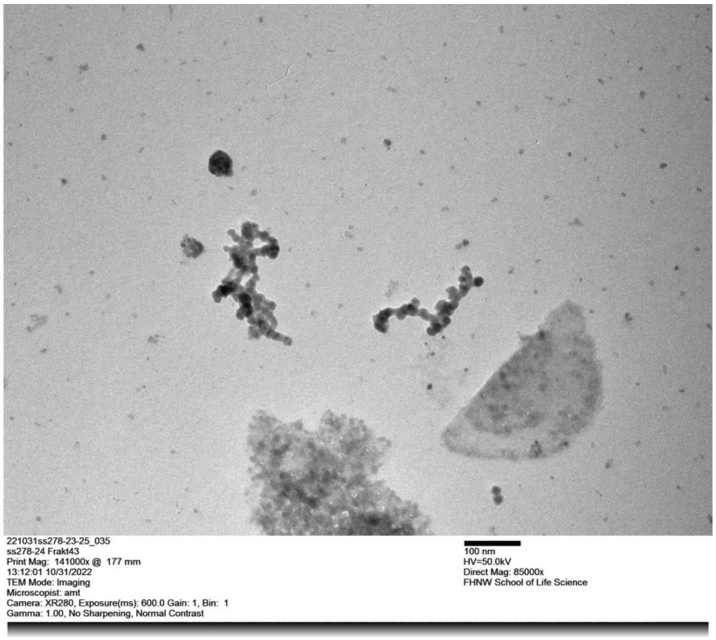

Figure SI-13: Transmission electron microscopy image of the SEC fraction nr. 43 of Form 1 digested by microwave, measured on a Copper/Formvar grid at 50kx with a Zeiss EM900

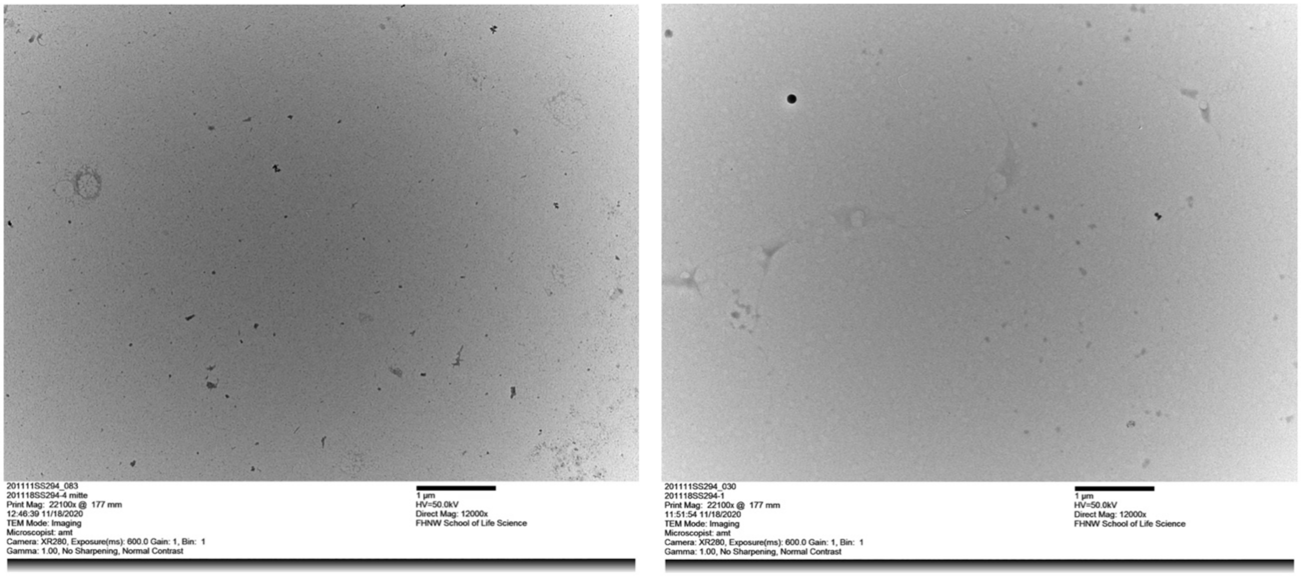

Figure SI-14: Transmission electron microscopy image of the premix with (left image) and without AA (right image) digested by Fenton agent and sample were incubated on a Copper/Formvar grid and rinsed with MQ-water, dried and measured at 50kx with a Zeiss EM900.

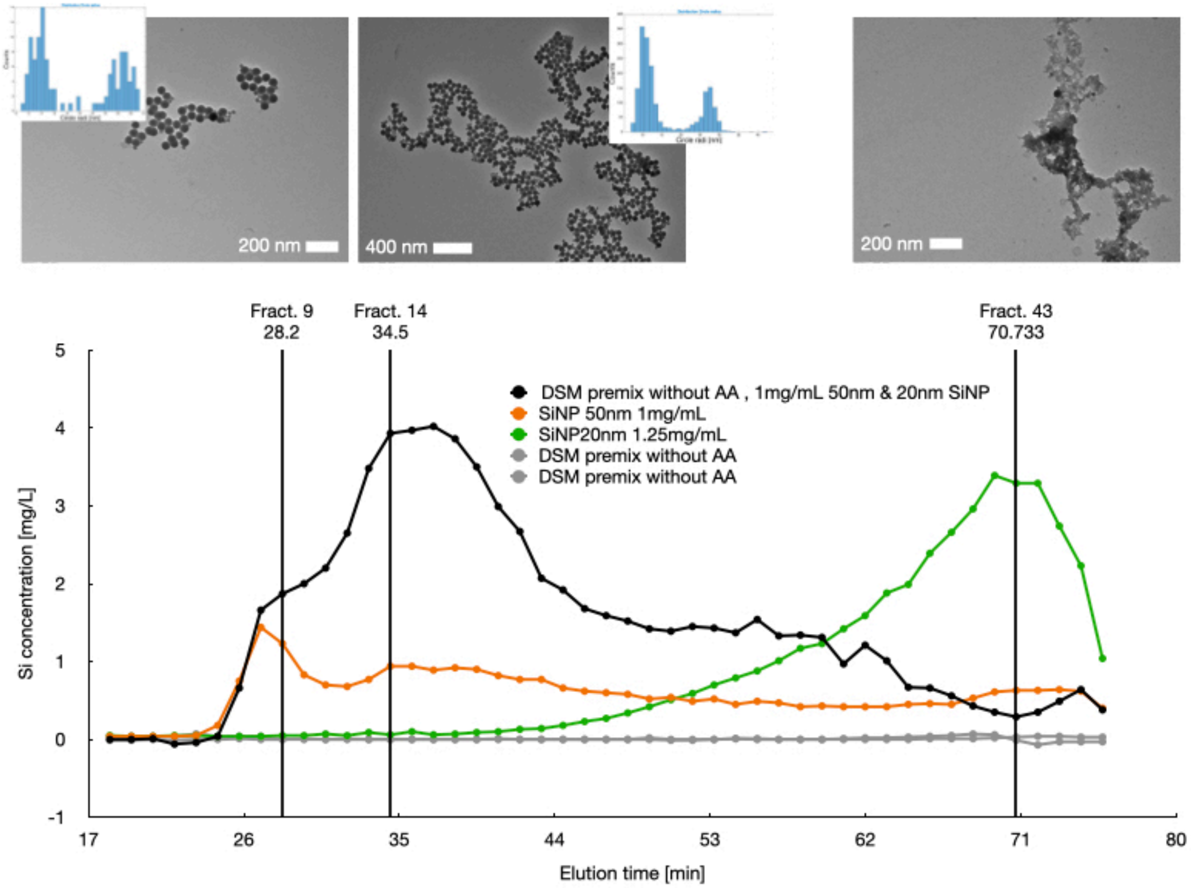

Figure SI-15: Size exclusion chromatogram of DSM premix sample without AA spiked with 1mg/mL 20 & 50nm SiNP standard samples (black) and the DSM premix sample without AA (grey). TEM images were taken from Fraction 9(left &middle) and 43(right) image, at 50kx with a Zeiss EM900

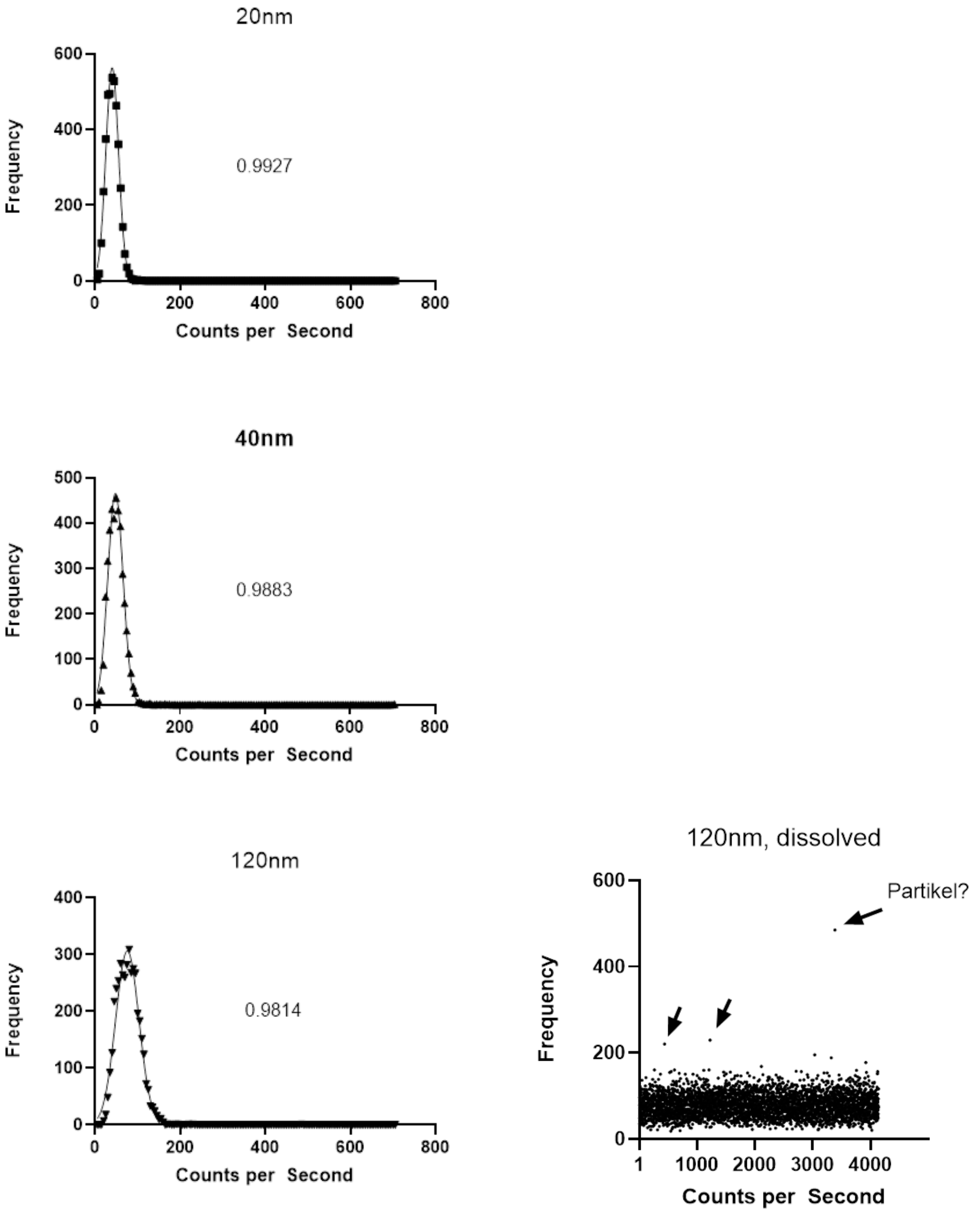

Figure SI-16: Preliminary single particle ICP-MS Measurement of SiNP standards with a diameter of 20, 40 and 120nm. The spikes at higher frequency were counted, spikes are marked with arrows in graph for the 120 nm on the right side. Counts went up for SiNPs with 120 nm but no significant frequency difference was observed for 20 and 40 nm. Measured with an Agilent ICP MS, samples were diluted to pg/L in MQ-water but concentration was lowered individually to count single particles.

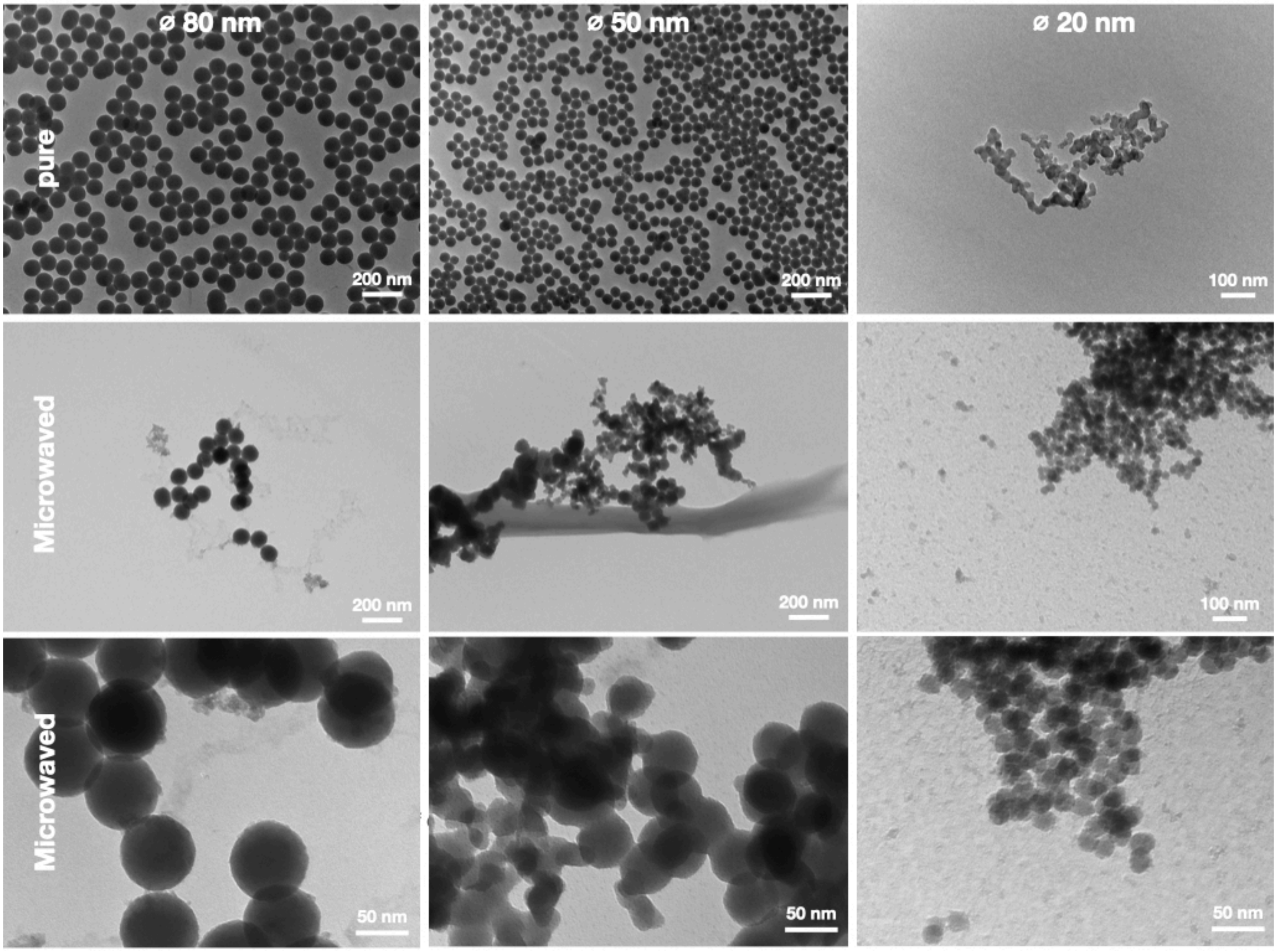

Figure SI-17: Transmission electron microscopy images of the standard SiNPs with 80, 50 and 20nm diameter (left to right column), at a magnification of 50kx pure (top row), at 50kx (middle row) and at 250kx (lowest row), after the microwave digestion with a Mars6 Microwave from CEM Corporation. Images were taken with a Zeiss EM900 on 200mesh carbon coated formvar copper grids after neutralisation.

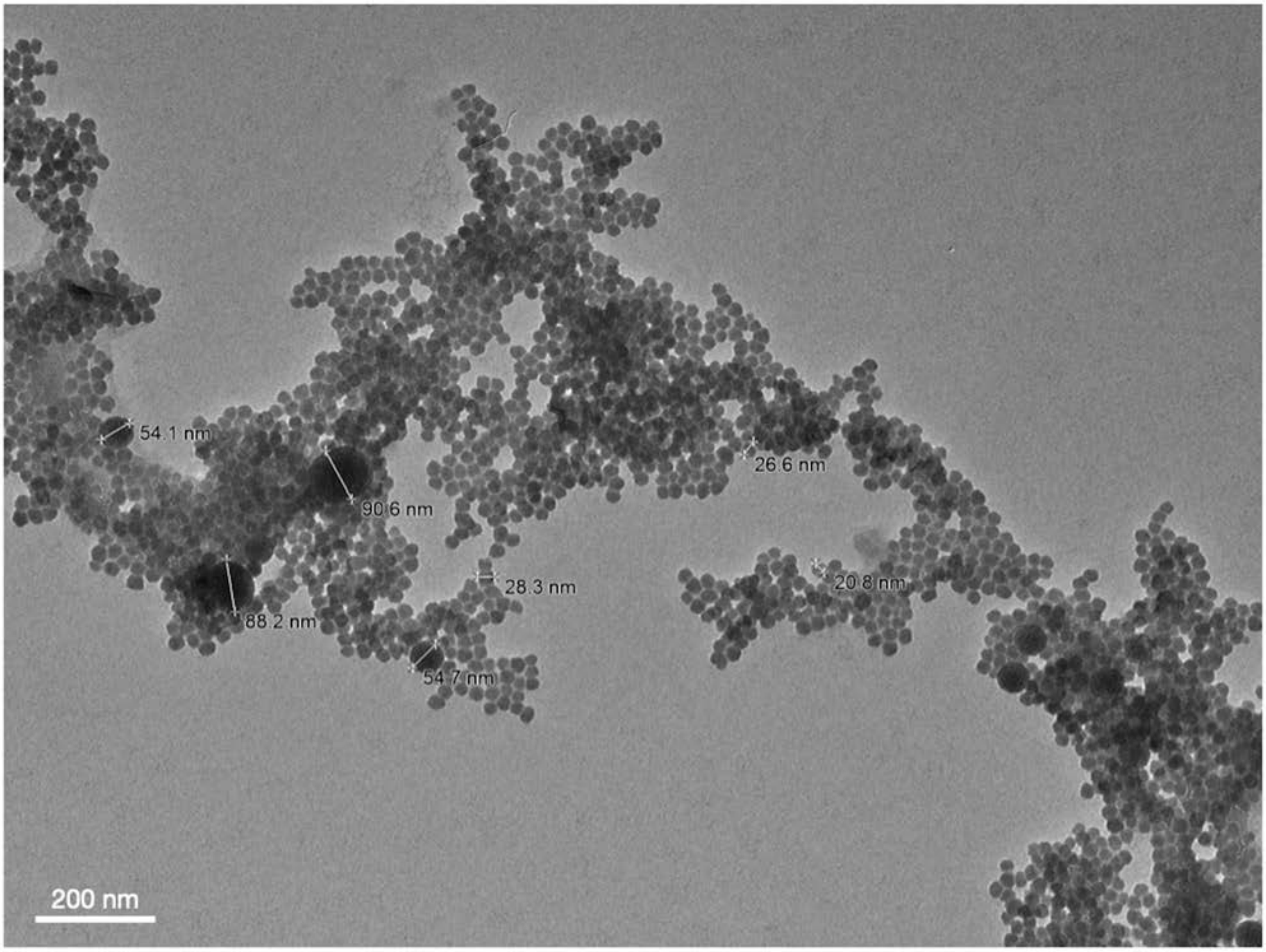

Figure SI-18: Cross contaminated fraction nr. 14 of a SEC run of SiNP standard with 20 nm diameter in PBS with 0.05% SDS as an eluent and Sepharose4B column. The column was not rinsed prior the run and the other standard SiNPs with 50 and 80nm diameter were run before. The cross contamination with the larger SiNPs is evident. (SS278-7)

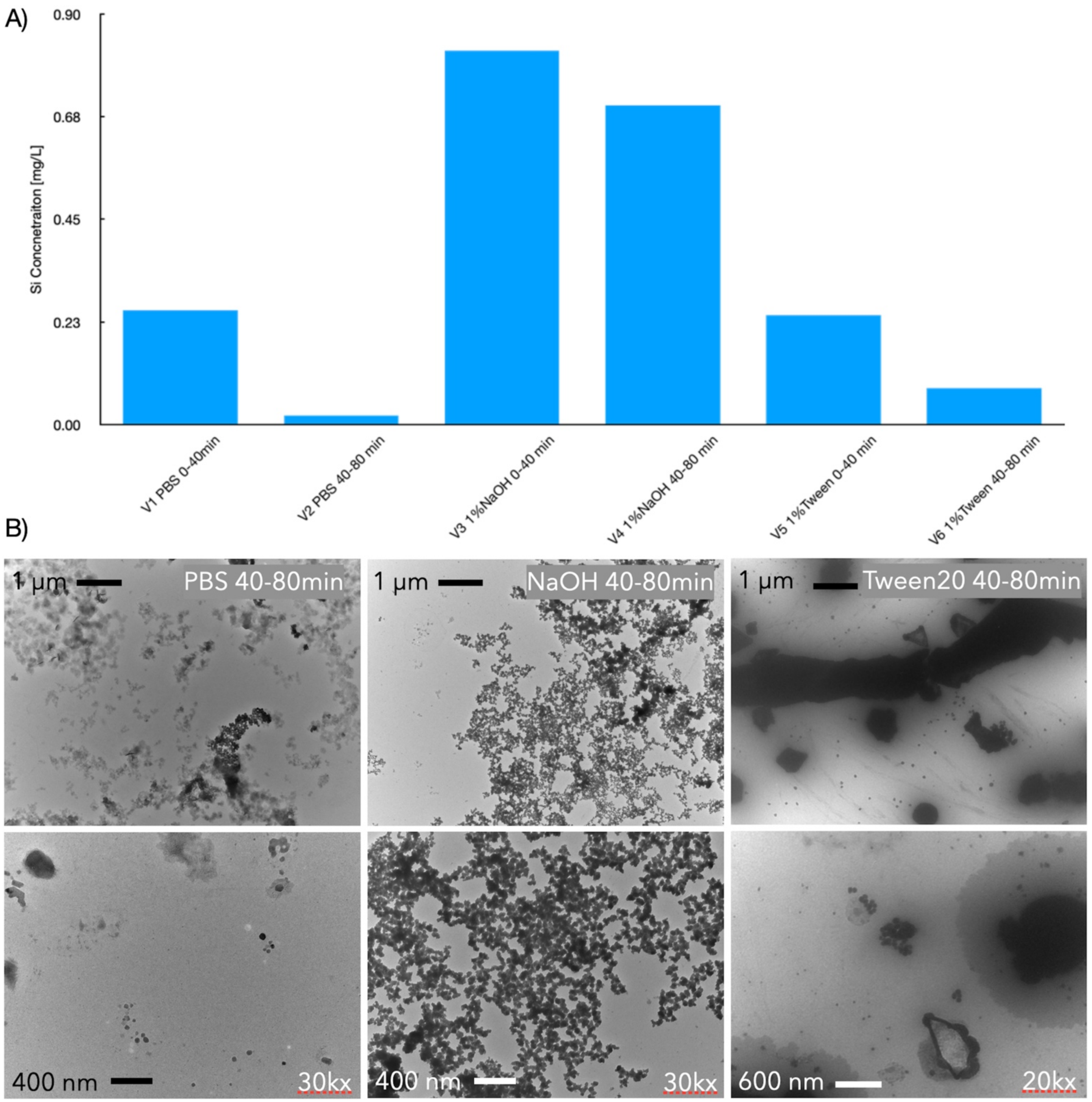

Figure SI-19: A) Silicon concentration in collected fractions of two SEC runs with three eluents; Phosphate buffered saline (PBS), PBS with 1% NaOH and PBS with 1% Tween20. For each eluent, the first SEC run (0-40min) was with injected standard SiNP 50nm and the second run was without sample injection (40-80min). SEC was done with a gravimetric Sepharose 4B column. The silicon content of the two fractions (0-40 and 40-80min) was measured by 4210 MP-AES from Agilent. B) TEM Images of the collected fractions of the second run (40-80min) of tall three eluents. Many particles were found for 1% NaOH/PBS the other two eluents showed lots of artefacts but not a lot of SiNPs. Images were taken with a Zeiss EM900 at 50kV, with the samples on 200 mesh Formvar coated copper grids.

MatLab "Find circle" script (Adapted from documentation; https://ch.mathworks.com/help/images/ref/imfindcircles.html)

```
clear all;clc;close all
%This allows you to spatially calibrate your image and then make distance or area measurements.

% Read in a standard MATLAB gray scale demo image.
% Read in the chosen image.
originalImage = imread('200827ss239-120nm_035.TIF');

RGB1 = cat(3, originalImage, originalImage, originalImage);
% Get the dimensions of the image.
% numberOfColorBands should be = 1.
[rows, columns, numberOfColorBands] = size(originalImage)
% Display the original gray scale image.
figureHandle = figure
imshow(originalImage, [])
axis on;
title('Original Image', 'FontSize', 16);
% Enlarge figure to full screen.
set(gcf, 'units','normalized','outerposition',[0 0 1 1]);
% Give a name to the title bar.
set(gcf,'name','Demo by ImageAnalyst','numbertitle','off')


[cx, cy, rgbValues, xi,yi] = improfile(1000);
% rgbValues is 1000x1x3. Call Squeeze to get rid of the singleton dimension and make it 1000x3.
rgbValues = squeeze(rgbValues);
distanceInPixels = sqrt( (xi(2)-xi(1)).^2 + (yi(2)-yi(1)).^2);
if length(xi) < 2
return;
end
% Plot the line (e.g. on scale bar).
hold on;
lastDrawnHandle = plot(xi, yi, 'y-', 'LineWidth', 2);
% Ask the user for the real-world distance.
userPrompt = {'Enter real world units (e.g. microns):','Enter distance in those units:'};
dialogTitle = 'Specify calibration information';
numberOfLines = 1;
def = {'nm', '1000'};
answer = inputdlg(userPrompt, dialogTitle, numberOfLines, def);
if isempty(answer)
return;
end
calibration.units = answer{1};
calibration.distanceInPixels = distanceInPixels;
calibration.distanceInUnits = str2double(answer{2});
calibration.distancePerPixel = calibration.distanceInUnits / distanceInPixels;
success = true;

message = sprintf('The distance you drew is %.2f pixels = %f %s.\nThe number of %s per pixel is %f.\nThe number of pixels per %s is %f',...
distanceInPixels, calibration.distanceInUnits, calibration.units, ...
calibration.units, calibration.distancePerPixel, ...
calibration.units, 1/calibration.distancePerPixel)
uiwait(msgbox(message));
%%
% Crop to picture to remove image information part
rect = [0, 0, 2000, 1425];
figure
J = imcrop(originalImage, rect);
imhist(J)

Gray= imadjust(J,[0 0.4],[0 1],[2]);

figure
imshow (Gray)

% Approximate circle radius (define an approriate value)
Rmin =6;
Rmax =10;

[centersDark, radiiDark] = imfindcircles(Gray,[Rmin Rmax], 'ObjectPolarity','dark','Method','PhaseCode', 'Sensitivity',0.96, 'EdgeThreshold',
0.3);

%shows circles in blue color
viscircles(centersDark, radiiDark,'EdgeColor',[0.8500 0.3250 0.0980], 'LineStyle','-','Linewidth',0.5);

% Counted circles:
Counts =length(radiiDark)

%Circle Radius in pixel
radiiDark;

%Circle Radius in nm
realradii = radiiDark*calibration.distancePerPixel

% Circle area in µm^2
area = (realradii).^2 *pi/1000

MeanRadius = mean(realradii)
RadiusStdev = std(realradii)
MeanArea = mean(area)
AreaStdev = std(area)

%Plot data 'circle area' as histogram
histogram(area,20)
ax.FontSize = 20
xlabel('Circle area [µm^2]', 'FontSize',20)
ylabel('Counts','FontSize',20)
title('\fontsize{30}\color[rgb]{0 .5 .8} Distribution Circle Area');
exportgraphics(ax,'Hist_CircleArea.jpg')
```
